# High-Throughput Detection of Cyanobacterial Form I Rubisco Assembly

**DOI:** 10.1021/acssynbio.5c00591

**Published:** 2025-12-22

**Authors:** Jackson W. Wysocki, ByungUk Lee, Tina Wang

**Affiliations:** Department of Chemistry, 5228 University of Wisconsin-Madison, Madison, Wisconsin 53706, United States

**Keywords:** Rubisco biogenesis, Chaperone−dependent assembly, Genetically encoded biosensor, RbcL/RbcS assembly, Protein engineering constraints

## Abstract

Rubisco catalyzes the CO_2_ fixation step in the dark reactions of photosynthesis. Transgenic expression of better-performing Rubisco orthologs in plants or discovery of improved mutants of Rubisco via protein engineering could theoretically accelerate plant growth and improve crop yields. However, efforts to heterologously express or engineer Rubisco are frequently stymied by the chaperone-dependent folding and assembly of the Rubisco holoenzyme, a process that can be disrupted by changes to Rubisco’s sequence. Elucidation of the effects that alterations to Rubisco’s sequence impose upon its biogenesis is hampered by reliance upon low-throughput methods for verification of Rubisco assembly. Here, we report the engineering of a genetically encoded biosensor to sense the assembly of Form I Rubiscos in *E. coli*. We show that the biosensor can detect the RbcS-dependent assembly of cyanobacterial Rubisco orthologs, the formation of chaperone-stabilized RbcL oligomeric assembly intermediates, and differences in assembly caused by mutations to the RbcL sequence. Additionally, we perform a large-scale examination of the relative assembly levels of a ∼7500-member *Halothiobacillus neapolitanus* RbcL mutant library by adapting the biosensor for use with phage-assisted noncontinuous selection. Our experiment predicts that the majority (>90%) of examined RbcL mutations exert a negative effect on assembly, lending support to the hypothesis that Rubisco biogenesis constrains both its natural evolution and improvement by protein engineering.

## Graphical abstract

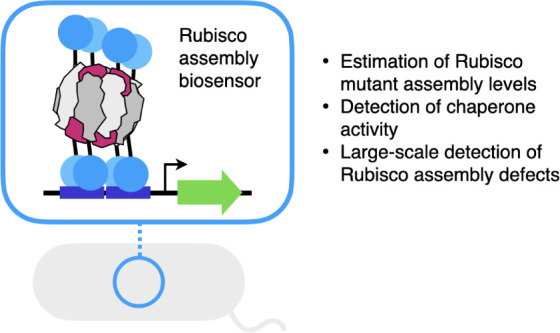

## Introduction

Ribulose-1,5-bisphosphate carboxylase oxygenase (Rubisco) catalyzes the CO_2_ fixation step in the Calvin Cycle. It is the most abundant enzyme on Earth but is notably slow (∼3–10 rxn/s) and erroneously reacts with O_2_ to generate a byproduct that must be recycled via energy-expensive photorespiration.^1^ Given its critical metabolic role in photoautotrophic organisms, Rubisco has been identified as an attractive target in efforts to increase photosynthesis and improve crop yields.^2,3^ Two general strategies have been pursued to this end: the first aims to directly improve Rubisco through protein engineering while the second seeks to replace the native Rubiscos in plants with more efficient orthologs from other species. So far, these efforts have not yielded improvements in plant growth, although several significant advancements have recently been reported.^4–23^

A complication for Rubisco engineering efforts lies in Rubisco biogenesis.^24^ Form I Rubisco, the structural form found in nearly all photoautotrophic organisms of significance (ex. land plants, algae, and cyanobacteria), is a hexadecameric complex composed of eight large subunits (RbcL) and eight small subunits (RbcS) (Figure 1a). For Form I Rubiscos, folding of the large subunit strictly depends on chaperonins, and holoenzyme assembly can require up to four chaperones.^24,25^ Mutating the Rubisco sequence in protein engineering efforts may exert deleterious effects on Rubisco’s inherent ability to fold and assemble as well as disrupt interactions with the chaperones that facilitate assembly. Stability and chaperone dependence are thus hypothesized to constrain both Rubisco’s natural evolution and artificial efforts to manipulate its activity.^13,26^ Similarly, many non-native Rubiscos experience diminished assembly when expressed in plants because of poor recognition by endogenous chaperones, posing a barrier to generating transgenic crops with more efficient Rubisco orthologs.^1,27^

**Figure 1:**
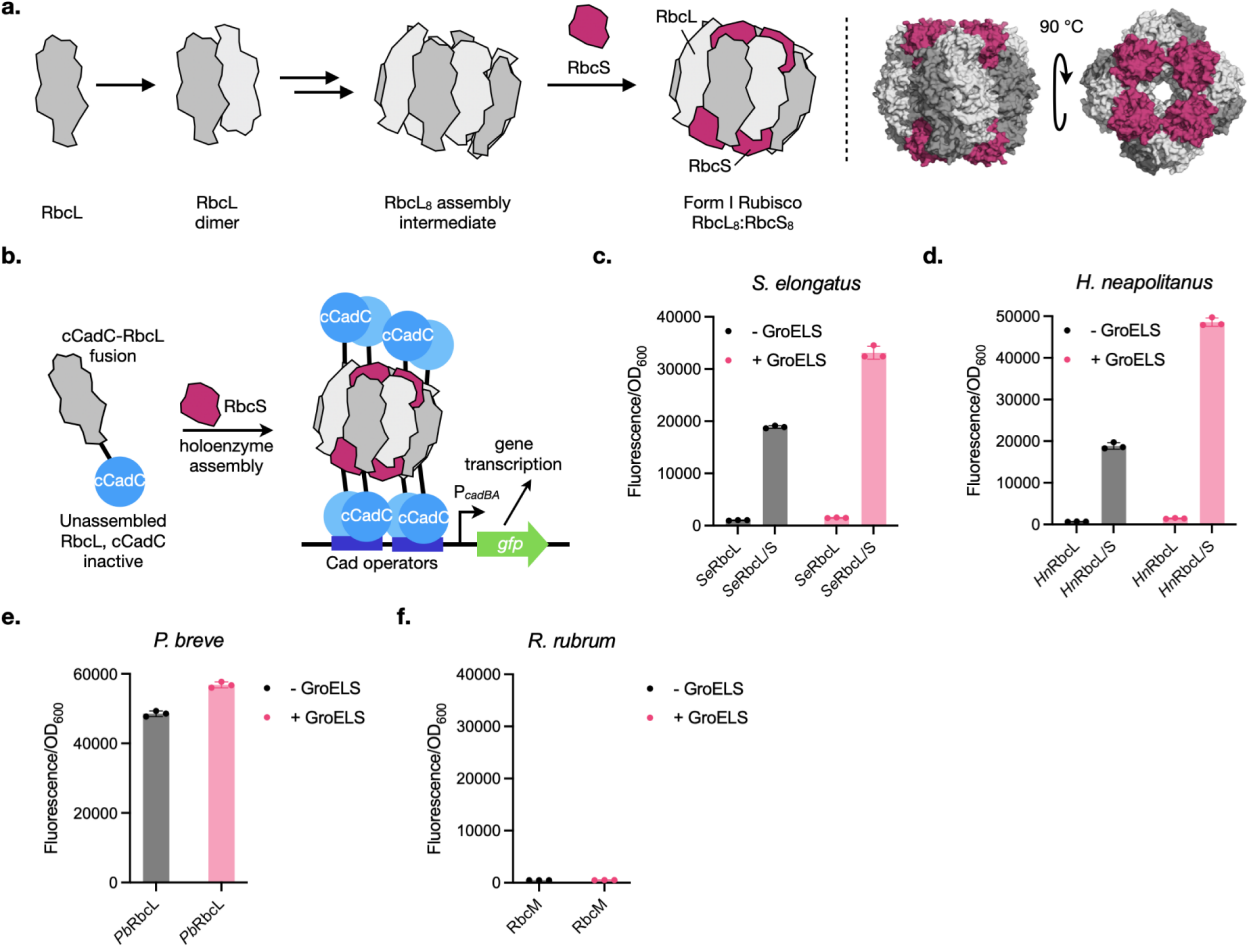
(a) Assembly and structure of Form I Rubisco. PDB accession code: 1RBL. (b) Overview of cCadC-based biosensor strategy for detecting Form I Rubisco assembly. The oligomerization-dependent transcription factor cCadC is inactive when fused to unassembled RbcL, but upon formation of an RbcL_8_S_8_ Rubisco complex, it is brought into proximity with other cCadC domains, enabling gene transcription of the *cadBA* promoter (P_*cadBA*_). (c–f) Ability of cCadC fusions to RbcL orthologs from (c) *S. elongatus* PCC6301, (d) *H. neapolitanus*, (e) *P. breve*, and (f) *R. rubrum* to activate *gfp* expression from P_*cadBA*_ in the absence (left) or presence (right) of GroELS overexpression. cCadC fusions were induced at 75 ng/mL aTc, and GroELS expression was induced at 0.1 mM IPTG. *Se*RbcL/S and *Hn*RbcL/S denote conditions where the corresponding RbcS was coexpressed.

It is not well understood how changes in the Rubisco sequence affect its assembly or recognition by chaperones, or what proportion of RbcL mutations may be deleterious to these processes. The Hayer–Hartl group demonstrated that RbcL mutations cause defects in chaperone-assisted assembly but not chaperonin-dependent folding; however, the scope of RbcL mutants characterized (∼500) was limited.^13^ More recently, the Savage group performed an exhaustive scan of all single point mutations of the Form II Rubisco from *Rhodospirillum rubrum* on its activity.^28^ However, this Rubisco is from a different structural class and comprised of a RbcL dimer (vs the Form I hexadecamer of RbcL and RbcS subunits); furthermore, the study did not directly assess assembly. A technical barrier limiting the large-scale characterization of sequence-assembly relationships is that methods enabling direct detection of Rubisco assembly are restricted to low-throughput measurements (such as native PAGE). While several strategies for assessing Rubisco activity in high throughput using engineered *E. coli* strains have been developed, they provide information on assembly only as a function of activity.^4,15,28–30^ Folding reporters (ex. split GFP^31^) have also been used to estimate Rubisco levels,^30^ but these only reflect RbcL solubility, which is not equivalent to the levels of fully assembled Rubisco: for example, chaperonin- and chaperone-bound intermediates also produce soluble RbcL.^32^

Here, we report a genetically encoded biosensor that can detect the formation of Form I Rubiscos from cyanobacteria, the most abundant photosynthetic organisms on Earth, in an *E. coli* model of Rubisco biogenesis. This strategy can report on the RbcS-dependent assembly of Rubisco orthologs from *Synechococcus elongatus* PCC6301 and *Halothiobacillus neapolitanus*, as well as the RbcS-independent assembly of the Form I’ Rubisco from *Promineofilum breve*. We also show that the biosensor can detect chaperone-stabilized RbcL assembly intermediates and predict the effects of point mutations in *H. neapolitanus* RbcL on relative levels of holoenzyme formation. Finally, we adapt the biosensor for use in phage-assisted noncontinuous selection (PANCS^33^) and demonstrate its utility in selecting assembly competent mutants from a library of over 7000 *H. neapolitanus* RbcL variants generated by site-saturated mutagenesis at three sequential residues. Our experiments suggest that the majority (>90%) of RbcL mutations examined led to decreases in holoenzyme assembly, but also that overexpression of a chaperonin (*E. coli* GroELS) could rescue assembly of a significant portion of these variants.

## Results and Discussion

### Development of a Biosensor for Form I Rubisco Assembly

Form I Rubisco is a hexadecamer made up of eight large subunits (RbcL) and eight small subunits (RbcS). The pathways by which cyanobacterial Form I Rubiscos are assembled have been worked out through extensive biochemical and structural investigations. After folding by chaperonins, RbcL associates into a series of assembly intermediates containing different numbers of RbcL dimers, with dedicated Rubisco chaperones able to assist in this process (*vide infra*). The final assembly intermediate is thought to comprise a RbcL octamer (RbcL_8_), which is then capped with RbcS subunits at either pole to form the Rubisco holoenzyme (Figure 1a).

We hypothesized that we could detect cyanobacterial Form I Rubisco formation by sensing the octameric oligomerization state of RbcL in the assembled holoenzyme. Recently, our group developed a transcription factor, cCadC, to detect homomeric protein–protein interactions in *E. coli*.^34^ cCadC is by itself monomeric but initiates transcription of its associated promoter P_*cadBA*_ when fused to a protein that self-interacts. Interestingly, while cCadC can be modestly activated by dimerizing proteins, it is significantly more active when fused to a high-order oligomer (trimers or greater). Thus, we hypothesized that by fusing cCadC to RbcL (cCadC-RbcL), we would be able to detect Form I Rubisco formation via a cCadC-RbcL_8_:RbcS_8_ complex driving expression of a reporter gene from P_*cadBA*_ (Figure 1b).

We chose the Form I Rubisco orthologs from cyanobacteria *Synechococcus elongatus* PCC6301 (*Se*Rubisco) and *Halothiobacillus neapolitanus* (*Hn*Rubisco) to test this strategy. The abilities of both orthologs to assemble in *E. coli* on their own (that is, without requiring dedicated Rubisco chaperones) are well-precedented.^32,35^ Fusions of cCadC to *S. elongatus* RbcL (*Se*RbcL) or *H. neapolitanus* RbcL (*Hn*RbcL) activated the transcription of an engineered GFP variant, mGreenLantern^36^ (hereafter referred to as “GFP”), from P_*cadBA*_ in a RbcS-dependent fashion (Figure 1c–d and Supplementary Figure 1a–b). Because RbcS addition is the last step of holoenzyme assembly, the requirement of coexpressed RbcS for cCadC-RbcL activity leads us to hypothesize that activation of our biosensor requires the formation of a cCadC-RbcL_8_:RbcS_8_ complex resembling the Form I Rubisco holoenzyme.

The assembly of Form I Rubisco occurs through oligomeric assembly intermediates, such as RbcL dimers and octamers (Figure 1a). Our inability to observe P_*cadBA*_ activation from cCadC fusions with *Se*RbcL and *Hn*RbcL in the absence of RbcS is consistent with reports that these Rubisco assembly intermediates are unstable.^37^ However, an alternative possibility is that cCadC does not detect oligomers comprised only of RbcL. To probe this, we fused cCadC to the octameric Form I’ Rubisco from *Promineofilum breve* (*Pb*RbcL) or the dimeric Form II Rubisco from *Rhodospirillum rubrum* (RbcM) and tested the ability of these constructs to activate P_*cadBA*_. Only cCadC-*Pb*RbcL produced an increase in GFP signal (Figure 1e–f and Supplementary Figure 1c), suggesting that octamers of the Rubisco large subunit can activate the biosensor while dimers do not. The inability to detect the RbcM dimer may be due to its head-to-tail arrangement placing the cCadC domains too far from each other to allow self-association.

### Effect of Rubisco Assembly Factors on Biosensor Activity

Rubisco biogenesis is highly chaperone dependent, and several assembly factors have evolved to assist in Form I Rubisco production.^37^ Group I chaperonins, such as GroELS in bacteria, are required to fold the large subunit in both Form I and Form II Rubiscos.^24^ To determine if our biosensor can detect the effects of chaperonins on Rubisco assembly, we overexpressed the *E. coli* GroELS chaperonin system, which increases the production of cyanobacterial Rubiscos in *E. coli*,^10^ in the presence of cCadC-RbcL fusions. GroELS overexpression roughly doubled the GFP signal produced by cCadC-*Se*RbcL and cCadC-*Hn*RbcL in a RbcS-dependent manner (Figure 1c). GroELS coexpression had no effect on cCadC-RbcM, even though it has been shown to increase expression of unfused RbcM.^38,39^

One convoluting factor in these experiments is that GroELS might also amplify the GFP signal from the biosensor by increasing GFP or cCadC expression. However, in control experiments we found that GroELS overexpression had no impact on GFP itself (Supplementary Figure 2a) and only increased signal from an unrelated cCadC fusion at high inducer concentrations (Supplementary Figure 2b).

After chaperonin-mediated folding, RbcL associates into oligomeric assembly intermediates whose formation can be assisted by additional chaperones.^24^ Two such chaperones are Raf1 and RbcX which complex with RbcL dimers and promote RbcL oligomerization before being displaced by RbcS (Figure 2a).^40–45^ The Raf1 and RbcX homologues from *S. elongatus* (*Se*Raf1 and *Se*RbcX) have both been shown to improve *Se*Rubisco assembly in *E. coli*.^46,47^ Thus, we hypothesized that coexpressing these chaperones might increase the fluorescence signal generated by the cCadC-*Se*RbcL fusion.

**Figure 2:**
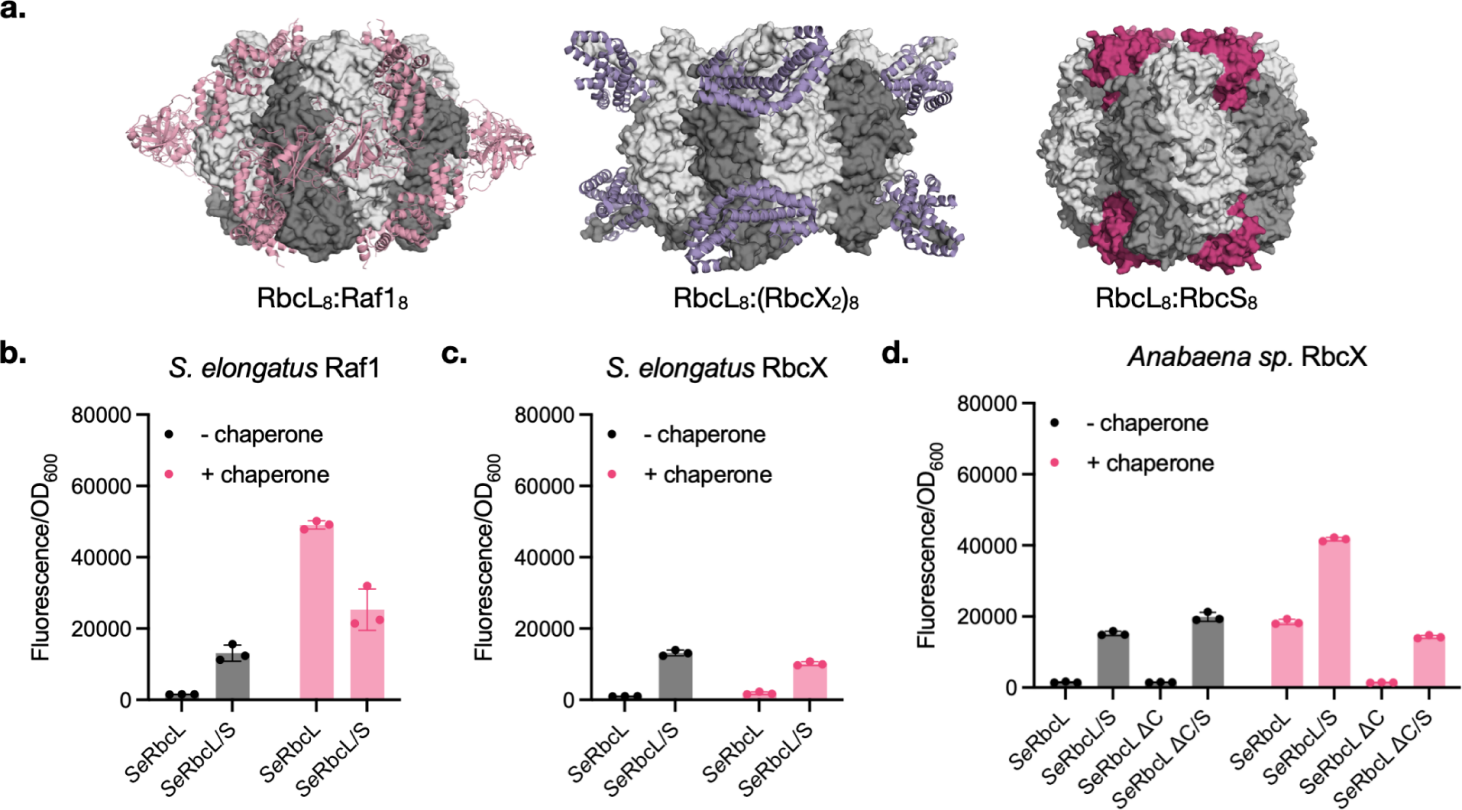
(a) Comparisons of the structures of a Raf1-bound RbcL_8_ assembly intermediate, an RbcX-bound RbcL_8_ assembly intermediate, and mature Form I Rubisco holoenzyme. PDB accession codes: 6KKM, 2WVW, and 1RBL. (b–d) Effect of coexpressing (b) *Se*Raf1, (c) *Se*RbcX, or (d) *Ana*RbcX on cCadC-*Se*RbcL-dependent transcription of *gfp* from P_*cadBA*_. Chaperones were expressed using an arabinose-inducible promoter (P_*BAD*_) from a plasmid that also encoded GroELS under the *tac* promoter (P_*tac*_). cCadC fusions were induced at 75 ng/mL aTc, and chaperone expression was induced with 7.5 μM arabinose. GroELS was not induced in this experiment. *Se*RbcL/S denotes coexpressed *Se*RbcS and ΔC indicates deletion of the last 12 residues in the *Se*RbcL C-terminus, which are critical for RbcX recognition.

We found that *Se*Raf1 greatly increases P_*cadBA*_ activation by cCadC-*Se*RbcL, to a greater extent than what is observed for cCadC-*Se*RbcL/RbcS (Figure 2b). Interestingly, this effect from *Se*Raf1 is reduced when RbcS is also supplied. The large signal increase in the absence of RbcS may suggest the formation of *Se*Raf1:*Se*RbcL oligomeric intermediates that are sufficiently stable to activate the biosensor. These complexes would not be expected to persist when RbcS is added and should instead convert to the RbcL_8_:RbcS_8_ holoenzyme,^32,43^ resulting in a reduction in fluorescence signal. This behavior is consistent with *in vitro* reconstitution experiments that show formation of *Se*Raf1:*Se*RbcL intermediates in the absence of RbcS and conversion to holoenzyme upon addition of RbcS. The origin of the higher signal from the *Se*Raf1:*Se*RbcL oligomers is currently unclear to us; one possibility is that *Se*Raf1:*Se*RbcL oligomers position the cCadC domains in a more optimal arrangement for P_*cadBA*_ activation compared to the cCadC-RbcL_8_:RbcS_8_ complex.

In contrast, *Se*RbcX coexpression had no effect on cCadC-*Se*RbcL or cCadC-*Se*RbcL/RbcS activation of P_*cadBA*_ (Figure 2c). A previous study could not reconstitute *Se*RbcX:*Se*RbcL complexes *in vitro* and instead employed a tighter-binding RbcX homologue from *Anabaena* sp. (*Ana*RbcX) (*K*_d_ ∼ 2 μM vs ∼105 μM for *Se*RbcX^40^) to produce RbcX:*Se*RbcL intermediates. We found that coexpression of *Ana*RbcX also generated a robust increase in cCadC-*Se*RbcL signal in both the presence and absence of RbcS (Figure 2d). The increase in GFP expression in the absence of RbcS implies the formation of a *Ana*RbcX:*Se*RbcL intermediate, while greater signal in the presence of RbcS suggests that *Ana*RbcX may either increase *Se*Rubisco assembly or form an *Ana*RbcX:*Se*RbcL:*Se*RbcS complex that triggers P_*cadBA*_ to a greater degree than *Se*Rubisco alone. Truncating the last 12 amino acids of *Se*RbcL, which contains the EIKFEFE motif recognized by RbcX, greatly reduces biosensor signal increases from coexpressed *Ana*RbcX (Figure 2d), consistent with previous findings that removal of this motif ablates RbcL assembly by RbcX.^40^ Together, these results suggest that our biosensor can also detect assembly intermediates formed between Rubisco chaperones and RbcL.

### Detection of the Effects of Point Mutations on Rubisco Assembly

Mutations in RbcL can impact Rubisco assembly, hampering efforts to improve Rubisco activity through protein engineering. Determining the relationship between Rubisco sequence, assembly, and activity remains an outstanding challenge. While our biosensor does not provide information on Rubisco activity, it could potentially yield insights on how RbcL mutations impact holoenzyme assembly. We engineered five *Hn*RbcL variants that bear single point mutations at the RbcL:RbcL, RbcL:RbcS, and inter-RbcL dimer interfaces that we hypothesized would perturb assembly (Figure 3a). Four of these, E102A, K139A, R208A, and E422A neutralize charged residues that are involved in salt bridges or other electrostatic interactions between subunit interfaces. Mutation of residues homologous to E102 or R208 in *Se*RbcL (E106/R212) has been shown to disrupt *Se*Rubisco assembly.^41^ An additional mutation, A136V, increases the size and hydrophobicity of an interfacial residue, which we hypothesized might interfere with higher-order oligomerization by increasing steric repulsion.

**Figure 3:**
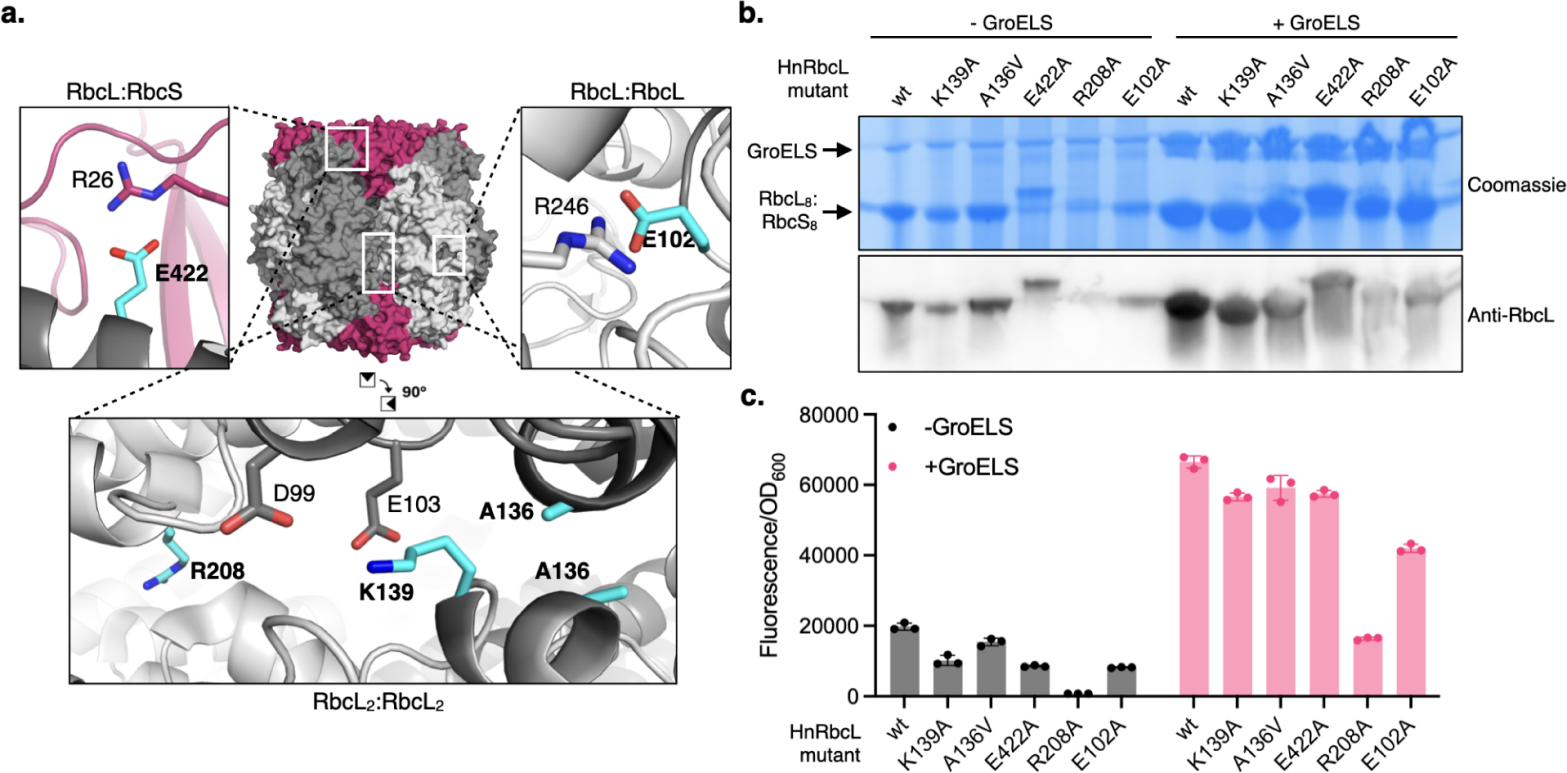
(a) Residues in *Hn*RbcL (colored in cyan and labeled in bold font) chosen for mutation to disrupt *Hn*Rubisco assembly. PDB accession code: 7ZBT. (b) Assembly of *Hn*RbcL mutants +/– GroELS overexpression measured by native PAGE and Western blotting against RbcL. See Supplementary Figure 3 for uncropped images. (c) Ability of cCadC-*Hn*RbcL mutants to activate *gfp* transcription from P_*cadBA*_ +/– GroELS overexpression. *Hn*RbcS was coexpressed in this experiment. cCadC fusions were induced at 75 ng/mL aTc, and GroELS expression was induced with 0.1 mM IPTG.

We first assessed the assembly of these *Hn*Rubisco mutants by native PAGE and Western blotting (Figure 3b). E102A, K139A, and E422A formed about half as much holoenzyme compared to wild-type *Hn*Rubisco. The E422A mutant exhibited decreased migration, suggesting that it may form a RbcL_8_ only assembly, consistent with this mutation disrupting the RbcL:RbcS interface. Of the remaining two mutants, R208A showed greatly decreased assembly while A136V assembled to similar levels as wild-type *Hn*Rubisco. Additionally, we coexpressed GroELS to determine its effect on the assembly of these mutants. All five mutants assembled to a greater degree when GroELS was overexpressed, consistent with the reported ability of chaperonins to improve production of destabilized proteins.^48^

We then cloned the same mutations into cCadC-*Hn*RbcL fusions and tested whether the differences in their assembly measured by native PAGE would translate into signal produced by our biosensor (Figure 3c and Supplementary Figure 4). In the absence of GroELS overexpression, we observed excellent agreement between Rubisco assembly measurements from our biosensor and native PAGE (Supplementary Figure 5a): cCadC-*Hn*RbcL E102A, K139A, and E422A mutants generated about half the amount of GFP fluorescence as wild-type *Hn*RbcL. A136V produced roughly the same signal as wild-type, while the R208A mutant failed to activate P_*cadBA*_. Upon GroELS overexpression, the fluorescence from both wild-type *Hn*RbcL and all examined mutants, including R208A, increased substantially (Figure 3c and Supplementary Figure 4). Under these conditions, our biosensor became less well correlated with *Hn*Rubisco assembly measured by native PAGE (Supplementary Figure 5b), potentially due to saturation of GFP signal, native PAGE band intensity, or both.

These results suggest that our biosensor can be used to measure differences in Rubisco assembly induced by changes to the RbcL sequence.

### High-Throughput Assessment of the Assembly of an RbcL Mutant Library

One motivation for developing the cCadC-RbcL biosensor was to provide a high-throughput way to assess Rubisco assembly: for example, in the context of a large library of RbcL variants. To enable this, we adapted our biosensor for use in a phage-assisted noncontinuous selection (PANCS^49^) format. PANCS and related techniques^50^ function by linking a desired phenotypic output from a gene of interest carried by filamentous bacteriophage M13 to its ability to replicate in *E. coli* host cells through the conditional expression of phage protein pIII (encoded by *gIII*), which is required for phage infectivity. Our strategy involved placing cCadC-RbcL/RbcS on the phage genome and putting *gIII* under the control of P_*cadBA*_. Thus, any phage encoding an assembly competent RbcL mutant would be able to reproduce on *E. coli* host cells by activating the cCadC dependent expression of pIII (Figure 4a).

**Figure 4:**
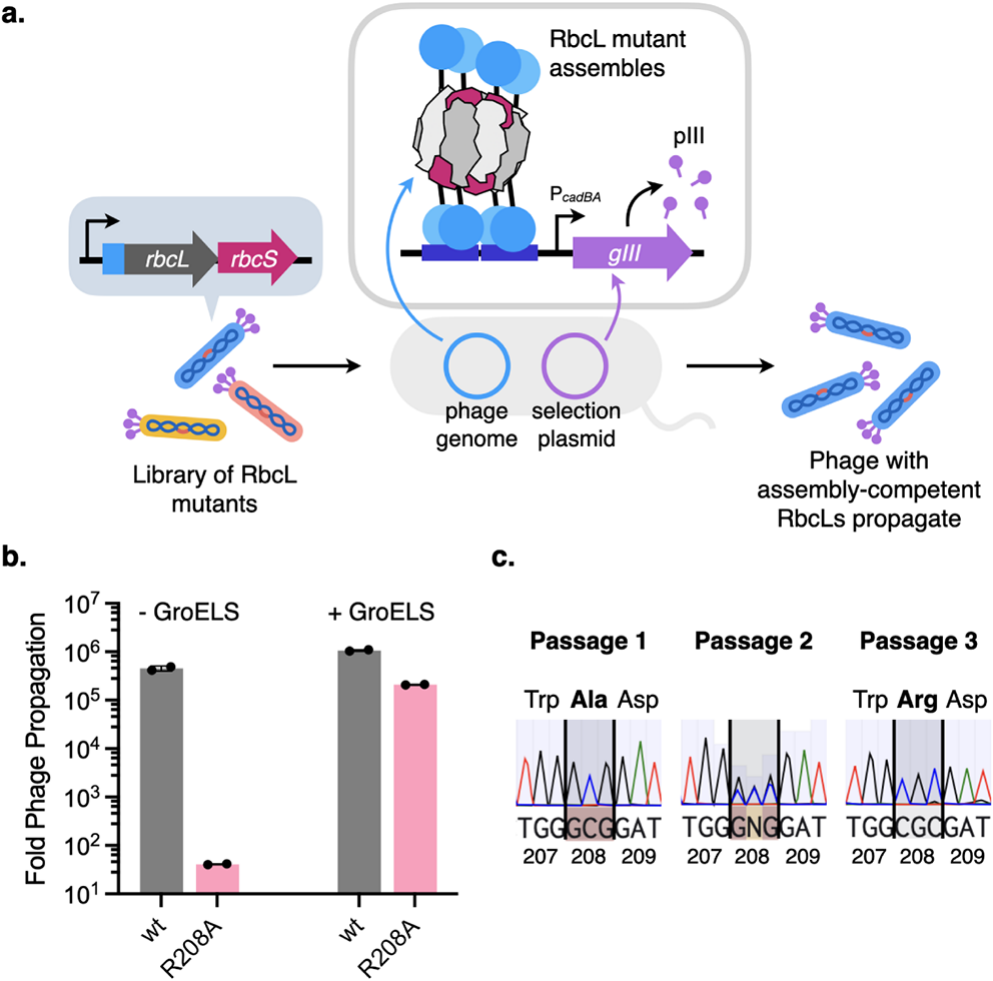
(a) PANCS strategy for assessing the assembly of RbcL variants on a large scale. A library of RbcL mutants fused to cCadC is encoded along with RbcS on engineered M13 bacteriophage that lack an essential phage protein pIII (encoded by *gIII*). These phage are used to infect *E. coli* host cells carrying a selection plasmid where *gIII* is provided under the control of P_*cadBA*_. Only phage that carry assembly-competent RbcL variants trigger *gIII* expression, enabling them to replicate further. (b) Propagation activity of phage encoding *Hn*RbcS and cCadC fusions to wild-type *Hn*RbcL or the assembly-deficient R208A mutant on host cells carrying a plasmid supplying *gIII* from P_*cadBA*_. GroELS was overexpressed from a separate plasmid in host cells. Fold propagation is calculated as the number of phage generated from an infected culture divided by the number of phage (10^5^) used to infect the culture. (c) Sanger sequencing chromatographs of the R208 locus of a phage pool consisting of a 1:1000 ratio of wild-type *Hn*RbcL to R208A mutant after selection on host cells carrying a P_*cadBA*_ *gIII* plasmid over three passages. After the first selection passage, the majority of phage carry the Ala (encoded by GCG) mutation at position 208, whereas by the third passage, the majority of the phage pool encode for wild-type *Hn*RbcL and show Arg (encoded by CGC) at position 208.

To benchmark the transition of our biosensor to a PANCS setting, we generated phage encoding cCadC fusions to wild-type *Hn*RbcL and the R208A mutant. These phage also coexpressed *Hn*RbcS in the same locus under a separate ribosome-binding site. Phage carrying the wild-type *Hn*RbcL sequence reproduced robustly on host cells bearing a plasmid supplying *gIII* from P_*cadBA*_, which is reflected by their high fold propagation (calculated as the number of phage generated from an infected culture divided by the number of phage used to infect the culture) (Figure 4b). In contrast, phage bearing the assembly deficient R208A mutant exhibited >1000-fold lower fold propagation, which could be rescued by overexpressing GroELS at the time of phage infection (Figure 4b). To simulate the enrichment of assembly competent RbcL library members, we performed a competition experiment where we passaged a 1000:1 ratio of R208A mutant phage to wild-type *Hn*RbcL phage on host cells with the P_*cadBA*_ *gIII* plasmid. Through each successive passage, we observed the wild-type *Hn*RbcL phage enriching over R208A phage through Sanger Sequencing of the R208 locus, with the wild-type sequence overtaking the phage pool in just three passages (Figure 4c).

Encouraged by these results, we sought to determine whether our selection could be used to assess *Hn*RbcL library for assembly competent variants. The *Hn*RbcL library was generated by site-saturation mutagenesis of D331/R332/A333, which lie immediately C-terminal to the RbcL loop 6 region (Figure 5a). Loop 6 closes over the Rubisco active site and is thought to contribute to protect reactive intermediates during catalysis.^1^ Several studies have examined the effects of mutating loop 6 and its surrounding residues on Rubisco’s catalytic properties.^51^ Residues D331–A333 are not interfacial and appear to be closer to the Rubisco surface; therefore, alterations to this region might be less detrimental to Rubisco assembly. Indeed, a recent deep mutational scan of RbcM revealed the homologous residues to be somewhat tolerant to mutation, especially at the analogous 332 and 333 positions.^28^ We hypothesized that examining the impact of diversifying this region on *Hn*Rubisco assembly might provide one snapshot of the constraints Rubisco biogenesis imposes upon engineering or evolving Rubisco for improved activity. Although the simultaneous mutation of three positions might be viewed as a large alteration to the RbcL sequence, Rubisco is widely considered evolutionarily trapped, suggesting that multiple concurrent mutations will be needed to significantly improve its activity.^13^ Thus, this degree of diversification is arguably not far removed from what may be necessary for a successful Rubisco protein engineering campaign.

**Figure 5:**
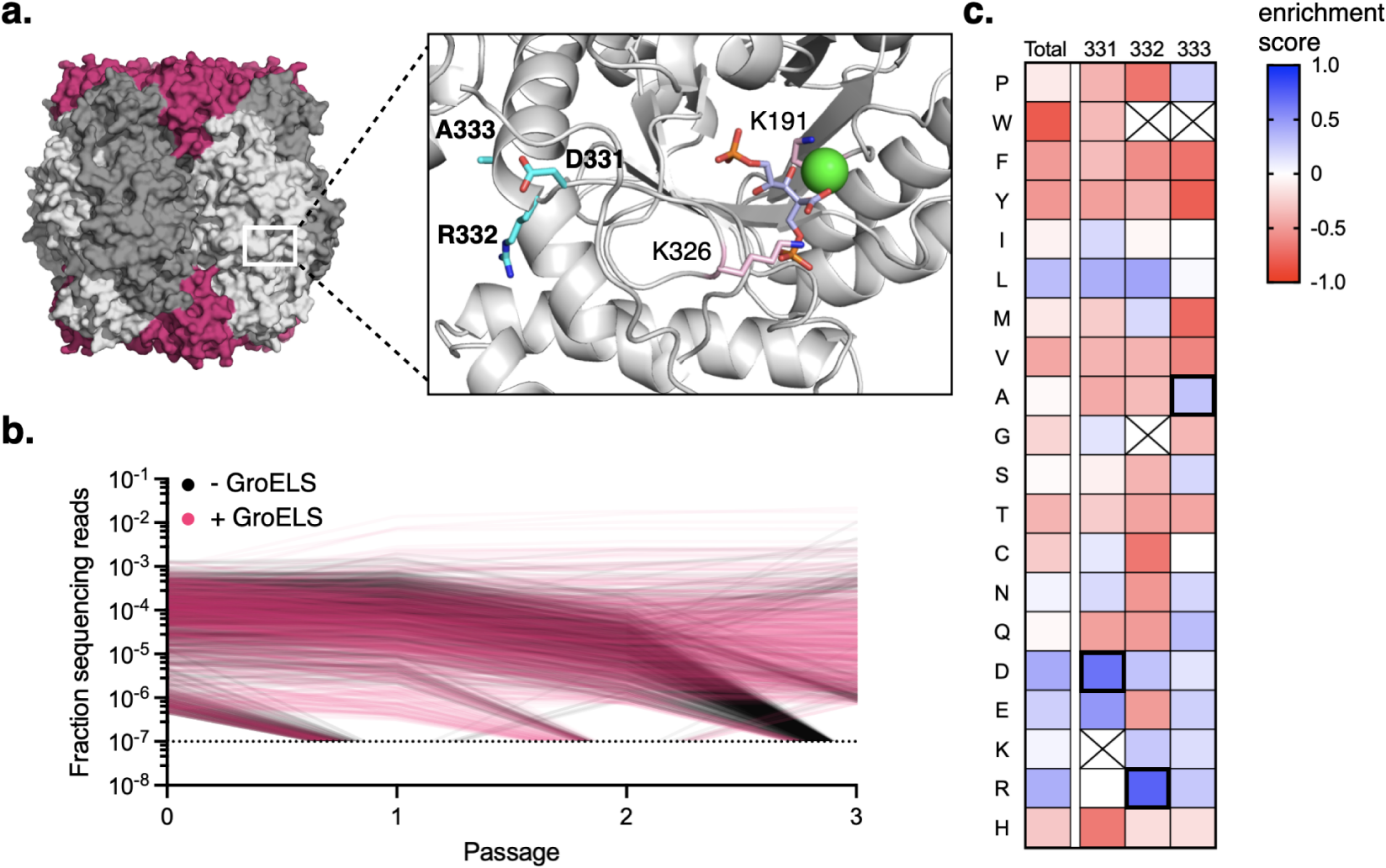
(a) Location of D321, R332, and A333 (in cyan) relative to the active site of *Hn*Rubisco. K326 and K191 are shown in pink. The inset was generated by alignment of an AlphaFold2 structure of *Hn*RbcL bearing a closed loop 6 conformation with a CABP- and Mg^2+^-bound *Se*RbcL structure. PDB accession code(s): 1RBL. (b) Trajectory of a random sampling of 1000 *Hn*RbcL variants over three selection passages with or without GroELS overexpression. *Y*-axis denotes the total fraction of sequencing reads corresponding to the variant, a representation of the relative abundance of each variant. The dotted line denotes the detection threshold. Variants that decline beyond the detection threshold are shown with their trajectories terminating at the dotted line. (c) Heat map of amino acids enriched at residues 331/332/333 in the 246 *Hn*RbcL variants with positive fitness scores in both +/– GroELS populations. The enrichment score for a given amino acid represents the log (base 10) of the ratio of all occurrences of a given amino acid in *Hn*RbcL mutants with positive fitness scores to all occurrences of the same amino acid in all 7547 starting variants. The total column shows overall enrichments for the indicated amino acid across all 3 positions. Boxes that are crossed out represent scenarios where 0 occurrences of that amino acid were detected in *Hn*RbcL variants with positive fitness scores at the given position. Sequences containing stop codons were excluded from this analysis.

We selected the D331/R332/A333 mutant library for assembly competent members under two conditions: one without and with additional GroELS expression from a plasmid borne by the host cells. After three rounds of phage passaging (Supplementary Figure 6), we characterized both the input library and the phage populations after every round using Illumina high-throughput sequencing. We tracked the overall trajectory of each of the 7547 variants (out of a total 9261 possible) that was detected in the input library with 10 or more sequencing reads (Figure 5b). Most variants (6117) were completely depleted after the third round of passaging in the population selected without GroELS induction, suggesting that many mutants have decreased assembly. However, this number was reduced drastically (to 1891) when GroELS was overexpressed. We also calculated a fitness score for each variant (Supplementary Tables 1 and 2). Positive fitness scores correspond to sequences that were enriched in the third passage relative to the starting pool, which we hypothesized should represent *Hn*RbcL mutants that assemble, while negative fitness scores indicate sequences that de-enriched in the third passage and should denote mutants with impaired assembly. Overall, ∼5.8% of the 7547 analyzed variants had positive fitness scores in the -GroELS condition. Inducing GroELS doubled this fraction of the population to ∼12%, suggesting that the GroELS chaperonin can play a significant role in rescuing *Hn*RbcL mutants with impaired folding or assembly.

We did not observe large differences in the most enriched (top 50) sequences between the – and + GroELS conditions (Supplementary Tables 1 and 2). The wild-type *Hn*RbcL sequence (“DRA”) was highly enriched in both populations, as expected. In addition, mutants with high sequence similarity to DRA (ex. DRQ, DLA, ERA, and DRN) also exhibited positive fitness scores and high enrichments. Variants with positive fitness appeared to favor negatively charged residues (D or E) at the first position (331) and positively charged residues (R or K) at the second position (332) (Figure 5c). Smaller (A, S, Q, N) or charged (D, E, K, R) residues were enriched at the third position (333). Several highly enriched sequences also contained proline at position 333, but sequences with proline at the first or second positions, or those that contained more than one proline, tended to have negative fitness scores. Finally, there was a general dearth of aromatic residues (H, F, Y, W) in highly enriched sequences.

We examined how accurately fitness scores from our *Hn*RbcL library selection predict assembly of the unfused *Hn*Rubisco variants. We selected seven mutants: three with positive fitness scores that diverge from the wild-type sequence (CRL, EKN, and MRQ), three mutants with negative fitness scores (MLL and PTQ), and one mutant that was completely purged by selection without GroELS (PSS). We also examined two mutants with a fitness score close to zero in the -GroELS population (LKS and ISP), implying these variants experienced neither strong enrichment nor de-enrichment.

In the absence of GroELS overexpression, *Hn*Rubisco variants with positive fitness scores all showed some degree of assembly when assessed by native PAGE, whereas variants with negative fitness scores were not observed to assemble (Figure 6). Additionally, GroELS increased assembly for all mutants but could not rescue MLL, PTQ, or PSS to wild-type levels of assembly. LKS and ISP, sequences with near-zero fitness scores in the -GroELS selection, also both assembled. These observations suggest that fitness scores close to zero do not accurately predict assembly efficiency. Thus, considering the sequences that were either not detected postselection or possessed a fitness score of −1.0 or lower, we estimate that approximately 90% of variants in the -GroELS population would be predicted to exhibit lowered assembly efficiency.

**Figure 6:**
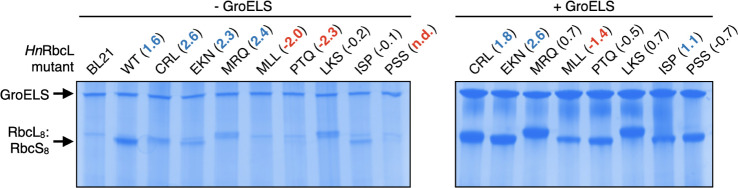
Assembly of *Hn*RbcL mutants identified from the library selection +/– GroELS overexpression measured by native PAGE. Fitness scores are provided in parentheses. “n.d.”, not detected in postselection population. See Supplementary Figure 7 for uncropped images.

### Caveats of the PANCS Strategy for High-Throughput Assessment of Rubisco Assembly

While all the *Hn*Rubisco variants we tested that possessed negative fitness scores showed greatly impaired assembly, none of the *Hn*Rubisco variants with positive fitness scores assembled to the same degree as wild-type *Hn*Rubisco in our native PAGE experiments (Figure 6, left). To shed some light onto potential causes, we tested the same variants in the GFP fluorescence assay (Supplementary Figure 8). Fluorescence values generated by the variants with positive enrichment scores were lower than that for wild-type *Hn*Rubisco (Supplementary Figure 8a) and showed agreement with the native PAGE results (Supplementary Figure 8b–c). This suggests that our PANCS experiment experienced low selection stringency enabling variants that, while still able to assemble, did not need to produce *Hn*Rubisco to the same level as the wild-type sequence to pass. To investigate further, we measured the propagation of phage carrying the set of *Hn*RbcL mutants tested in Figure 3 (Supplementary Figure 9). We observed that, while phage encoding the assembly deficient R208A mutant exhibited a large impairment in propagation, phage encoding mutants that produced large decreases in fluorescence in the GFP assay experienced only modest reductions in propagation.

The GFP assay also revealed that the CRL and EKN mutants produced less fluorescence compared to the MRQ mutant, despite all three of these variants showing highly similar fitness scores in the PANCS experiment. In addition, the CRL and EKN sequences were strongly enriched in the -GroELS selection, representing ∼46% and ∼5% of the total population, respectively (Supplementary Table 1). To investigate this discrepancy, we characterized the full RbcL sequence of 16 randomly sampled phage in the postselection pool through Sanger Sequencing. Seven phage encoded the CRL variant. Notably, all seven carried an additional mutation, G165S, at a region we did not mutagenize. One phage encoded the EKN variant, which also coincided with an additional mutation, W338L, once again occurring outside of the region on RbcL targeted for site-saturation mutagenesis. The other 8 phage only encoded mutants at D331/R332/A333.

When tested in the GFP assay, CRL/G164S and EKN/W338L produced markedly increased fluorescence, at the same level or higher than that of wild-type *Hn*RbcL (Supplementary Figure 10). This suggests that the very strong enrichment of the CRL and EKN sequences was likely due to the coincidence of these additional mutations that may have spontaneously arisen during library cloning. The *Hn*RbcL W338 residue corresponds to F342 in *Se*RbcL; mutations to Leu at this position increase *Se*Rubisco assembly in *E. coli* and have been frequently observed in directed evolution experiments with *Se*Rubisco using the Rubisco-dependent *E. coli* (RDE) system. Thus, it is possible that the W338L mutation in *Hn*RbcL increases *Hn*Rubisco assembly in a similar fashion. It is currently unclear to us how the G164S mutation may be impacting *Hn*Rubisco assembly.

Together, these experiments suggest that, while the PANCS strategy provides a reasonable estimation of whether a Rubisco mutant will lose the ability to assemble, in its present form it can only give a qualitative assessment of Rubisco assembly. The selection stringency needs to be further optimized to separate out mutants with wild-type levels of assembly from those that retain assembly but with some degree of impairment. Furthermore, mutations that spontaneously occur elsewhere in RbcL can convolute PANCS results; long-read sequencing may thus be required to control for input library quality (as was performed in ref 28) or used to more thoroughly characterize selection outcomes.

## Conclusion

We have developed a biosensor strategy to assess cyanobacterial Form I Rubisco assembly in high throughput in *E. coli*. Our strategy can detect the assembly of the Form I Rubiscos from *S. elongatus* PCC6301 and *H. neapolitanus* as well as the assembly of the Form I’ Rubisco from *P. breve*. Using the biosensor, we examined the effects of RbcL mutations on *H. neapolitanus* Rubisco assembly on large scale by selecting a site-saturation library of three positions in *H. neapolitanus* RbcL for variants that retained the ability to assemble. Despite choosing a RbcL region distal to intersubunit interfaces, > 90% of variants were predicted by our experiment to exhibit diminished assembly, highlighting the constraints that folding and assembly place upon Rubisco evolution. Characterization of the individual variants by native PAGE suggests that fitness scores are reasonable predictors of whether a given variant can assemble. We also found that GroELS overexpression compensates for assembly defects to a significant degree and roughly doubles the number of variants that survived our selection. These observations are consistent with studies demonstrating that chaperonins can “buffer” the deleterious effects mutations can impose on protein folding and stability during evolution generally,^48^ and that GroELS overexpression can expand the diversity of *Se*Rubisco variants undergoing directed evolution in the RDE system.^13^

Our system is subject to caveats in addition to the ones already discussed (*vide supra*). The scope of Rubiscos we examined in this study is limited and exclusively of prokaryotic origin. Eukaryotic Rubiscos have more demanding assembly requirements. A coinciding study from our group has found that our strategy can detect chaperone-bound assembly intermediates of Rubiscos from the dicots *A. thaliana* and *N. tabacum*,^52^ but how our biosensor might function with other eukaryotic homologues remains to be seen. Additionally, our method uses fusions to RbcL which may perturb the structure of assembled Rubiscos, and while our strategy appears to function well in determining relative assembly levels between mutants of the same Rubisco, it is not reliable for assessing relative assembly between different Rubiscos. Finally, because cCadC only requires oligomerization to be active, stable assembly intermediates or mutations that create non-native RbcL oligomers will turn on the biosensor. Further work is needed to determine to what extent signal from such oligomeric assemblies will convolute results obtained from the biosensor. Thus, care should be taken when examining a new Form I Rubisco or mutagenizing the Rubisco sequence.

In addition to facilitating the study of Rubisco biogenesis, the ability to monitor Rubisco assembly in high throughput may prove helpful to Rubisco engineering. For example, the ability to preselect for Rubisco mutations that preserve assembly may enhance directed evolution efforts to improve Rubisco activity by purging libraries of nonassembled (and therefore inactive) members. The results of our selection of *Hn*RbcL mutants suggest that assembly impaired variants can constitute a large fraction of mutant libraries. On the other hand, Rubisco selections often discover variants that increase apparent activity by boosting the amount of Rubisco produced.^10^ In the past, these mutants have been identified by laborious counter-screening;^7^ our biosensor may offer a faster way to eliminate them. Indeed, one such mutation (F345L) that frequently arises in directed evolution experiments using *Se*Rubisco also spontaneously arose and was enriched in our selection with *Hn*Rubisco (W338L).^7^ Finally, our biosensor may streamline efforts to identify chaperones that promote heterologous Rubisco assembly. This might be especially advantageous if applied to Rubiscos targeted for transgenic expression in plants, where chaperone compatibility is a significant barrier to achieving high levels of heterologous Rubisco production.

## Supplementary Material







## Notes

Experimental materials and methods; (1) GFP fluorescence from cCadC–RbcL fusions; (2) effect of GroELS overexpression on biosensor components; (3) uncropped gels; (4) GFP fluorescence from cCadC fusions to *Hn*RbcL mutants; (5) relationship between biosensor GFP signal and *Hn*Rbc assembly levels from native PAGE; (6) overview of *Hn*RbcL library selection scheme; (7) uncropped gels; (8) assembly of *Hn*RbcL variants from PANCS measured by biosensor vs native PAGE; (9) propagation of phage encoding *Hn*RbcL mutants; (10) effects of additional mutations on cCadC fused to *Hn*RbcL variants from PANCS (Supplementary Figures 1–10); plasmids, primers, reagents, and equipment used in this work (Supplementary Tables 3–5); Python scripts used to analyze high-throughput sequencing of the *Hn*RbcL library selection (Supplementary Note 1) (PDF)
Results from *Hn*RbcL library selection (Supplementary Tables 1 and 2) (XLSX)
GenBank sequences of plasmids used in this work (ZIP)
