# Supplementary material for: High-Throughput Detection of Cyanobacterial Form I Rubisco Assembly
Source: ACS Synth Biol. 2025 Dec 22;15(1):161–70. doi: 10.1021/acssynbio.5c00591 (PMC12814518; doi:10.1021/acssynbio.5c00591)
Supplement: Supplementary file 1 [file sb5c00591_si_001.pdf]

Supplementary Information for

High-throughput measurement of cyanobacterial Form I Rubisco biogenesis

Jackson W. Wysocki,[1] ByungUk Lee,[1] and Tina Wang[1,*]

[1] Department of Chemistry, University of Wisconsin-Madison, Madison WI, 53706
* Correspondence should be addressed to Tina Wang: twang495@wisc.edu

Materials and Methods
Supplementary Figures 1-10
Supplementary Tables 3-5
Supplementary Note 1
Supplementary References

Materials and Methods

General Methods:

The following working concentrations were used for antibiotics (Gold Biotechnology): ampicillin, 50 μg/mL; chloramphenicol, 25 μg/mL; kanamycin, 50 μg/mL; and spectinomycin, 100 μg/mL. Nuclease-free water (Omega Bio-Tek) was used in PCR reactions, cloning, and transformations. For other experiments, water purified by MilliQ purification system (Millipore). Q5 and Q5U High-fidelity polymerases (New England Biolabs). USER Enzyme (New England Biolabs) used for USER cloning and T4 Ligase, T4 PNK, and DpnI (New England Biolabs) used in blunt end cloning to install single mutations. A full list of plasmids used in this work is given in **Supplementary Table 3**. Key primer sequences are listed in **Supplementary Table 4**. A full list of reagents and equipment used in this work is given in **Supplementary Table 5**.

E. coli strain S2060[1] was used for plasmid cloning. A strain lacking endogenous *cadABC* (S2060 Δ*cadABC*) was generated using Lambda Red recombineering and used for all experiments employing cCadC, unless otherwise noted. *E. coli* strain BL21 DE3 was used to express unfused Rubiscos for native PAGE and western blotting experiments. *E. coli* strain DH10β (New England Biolabs) was used for phage library cloning and strain S2208[1] for plaque assays for phage titer determination. Bacterial transformations were performed using the TSS method. 2xYT (US Biological) or Davis Rich Media (DRM; US Biological) were used for culturing bacteria.

Plasmid cloning:

Plasmids were cloned by USER assembly or blunt-end cloning. DNA sequences for *S. elongatus* PCC 6301 *rbcL* and *rbcS,* were amplified from rbcLS-pMal-2px (Addgene 41621) and the DNA sequences for *H. neapolitanus rbcL*, *rbcS*, and *raf*2 (*acRAF*) were amplified from pCCM' (Addgene 162709) and pCB' (Addgene 162703). Other DNA sequences were obtained as gene fragments from Twist Biosciences. Plasmids used in this study are listed in **Supplementary Table 3**. Plasmid maps can be found in **Supplementary File 1**.

Protein gel electrophoresis and western blotting:

Single colonies of BL21 DE3 *E. coli* transformed with Rubisco expression plasmids were inoculated into 2 mL of 2xYT media containing maintenance antibiotics and incubated overnight at 37 °C with shaking. Saturated overnight cultures were diluted 1:100 into 6 mL of 2xYT containing maintenance antibiotics. Once the OD_{600} reached 0.6 – 0.8, the culture was induced with 1 mM IPTG and incubated at 30 °C with shaking for a further 18 h. 3 mL of this culture was at 8000 rcf for 4 minutes. Pellets were resuspended in 150 μL lysis buffer (B-PER (ThermoFisher) supplemented with 10 mM $MgCl_2$, 1 μg/μL lysozyme, and 1 mM DTT) and incubated on ice for 25 minutes to lyse. The lysate was then centrifuged at 4 °C at 20,000 rcf for 25 minutes. The soluble lysate was collected and protein concentration estimated by Bradford assay. 10 μg of soluble protein was loaded on a 7.5% TGX protein gel (BioRad) for **Figure 3** or a hand-cast 6 % polyacrylamide gel for **Figure 6**. Proteins were separated at 140 V for 20 min followed by 170 V for 240 min at 4 °C in native PAGE running buffer (25 mM Tris pH 8, 190 mM

glycine). Gels were stained with ReadyBlue Protein Gel Stain (Sigma-Aldrich) and imaged with a BioRad Gel-Doc EZ Imager. Densitometry analysis was performed using ImageJ.

For western blotting, proteins were transferred to PVDF membranes (Biorad) at 100 V for 60 min in native PAGE running buffer with 20% v/v methanol at 4 °C. Membranes were blocked with 3% m/v bovine serum albumin (BSA) in Tris-buffered saline (TBS) for 1 hour at room temperature. Then, the membrane was incubated with a 1:1000 dilution of rabbit α-rbcL primary antibody (Agrisera AS03 037) in TBS overnight at 4 °C, followed by three washes with TBST (TBS + 0.1% v/v TWEEN-20). The membrane was then incubated with horseradish peroxidase-conjugated goat α-rabbit secondary antibody (Azure AC2114), followed by three washes with TBST. Finally, the membrane was treated with Radiance Q chemiluminescence substrate (Azure) and imaged using an Azure c400 imager.

Fluorescence assays:

Unless noted otherwise, all fluorescence assays were carried out using strain S2060 *ΔcadABC*. The P_{cadBA} *mGreenLantern* reporter plasmid pSL012 was co-transformed with plasmids encoding the cCadC fusion of interest either alone or with a chaperone-expressing plasmid. Single colonies were inoculated into 2xYT media containing maintenance antibiotics and incubated overnight at 37 °C with shaking. Overnight cultures were diluted 1:100 into DRM containing maintenance antibiotics in a 96 deep-well plate and incubated at 37 °C with shaking until the OD_{600} reached ~0.4 before constructs were induced. The induced cultures then incubated overnight at 30 °C with shaking for 17 h. Then, 100 μL of culture was transferred to a 96-well black clear-bottom plate. Fluorescence (485 nm excitation; 544 nm emission) and OD_{600} were measured using a Tecan Infinite M Plex plate reader. OD normalized fluorescence values were calculated by dividing raw fluorescence signal by background-subtracted OD_{600}.

Phage cloning:

USER assembled phage genomes were transformed into strain S2208 and allowed to outgrow overnight in 8 mL 2xYT media (no antibiotics) at 37 °C with shaking. The next day, 1.4 mL of the saturated culture was centrifuged at 8000 rcf for 2 min and the phage-containing supernatant isolated via plaque assay as described previously.[2] Individual plaques corresponding to monoclonal phage were inoculated into 2xYT media and incubated overnight 37 °C with shaking. The saturated culture was centrifuged to isolate phage, which were verified by Sanger Sequencing.

To clone the phage-encoded *H. neapolitanus rbcL* library, phage spJW001a was amplified using primers JW0112/JW0113 and primers AB019/AB020. The resulting two amplicons were assembled by USER assembly and the product transformed into DH10β *E. coli* containing pJC175e. After 2 h outgrowth in 2xYT media (no antibiotics) at 37 °C with shaking, the library was titered via plaque assay to estimate library size. The outgrowth was allowed to continue overnight. Phage was harvested as described above and then further amplified by infecting a 100 mL culture of S2208s grown to mid-log in DRM with maintenance antibiotics. After 6 h, 1 mL of culture was removed and the phage were isolated and titered by plaque assay.

Phage propagation experiments:

S2060s *ΔcadABC* host cells transformed with pJH048 (P_{cadBA} *gIII*) and pJW046 (P_{BAD} *groELS*) plasmids were grown from 1:100 back-diluted overnight cultures in 2 mL DRM containing maintenance antibiotics until they reached OD_{600} ~ 0.4 - 0.6. Host cells were infected with 10^5 pfu of the phage of interest and, where appropriate, *groELS* expression induced with 0.1 mM arabinose. Cultures were incubated at 37 °C with shaking for 18 h. Phage were harvested and titered as described above.

<u>*Phage Library Selection:*</u>
A single colony of S2060s *ΔcadABC* host cells transformed with pJH048 and pJW046 was inoculated in 5 mL 2xYT containing maintenance antibiotics and grown at 37 °C with shaking overnight. The next day, 2 mL of the resultant saturated culture was added to 200 mL DRM containing maintenance antibiotics and incubated at 37 °C with shaking. Once the OD_{600} reached ~0.5, cultures were infected with 10^7 pfu of the phage library. For the +GroELS condition, 0.1 mM arabinose was added at the time of phage infection to induce GroELS expression. Phage were allowed to propagate for 18 h at 37 °C with shaking. Cultures were then centrifuged for 20 min at 4000 rcf at 4 °C. The phage-containing supernatant was collected and used to infect subsequent passages. For these subsequent passages, overnight cultures of host cells were diluted 1:100 into 150 mL DRM containing maintenance antibiotics. Once the OD_{600} reached ~0.6 – 0.7, 50 mL of phage harvested from the previous passage were used to infect the culture and arabinose was added to induce GroELS in the +GroELS population. Phage were allowed to propagate for 18 h at 37 °C with shaking, after which phage were harvested as described above. The number of phage in each passage were estimated by plaque assay.

<u>*Next Generation Sequencing:*</u>
Phage pools were first PCR amplified with JW0117 and JW0118 (28 cycles). Amplicons were purified and used as template in a second PCR with index primers (32 cycles). The products were gel-extracted and pooled. High-throughput sequencing was performed by the University of Wisconsin-Madison Biotechnology Center (UWBC) using a shared sequencing service on the NovaSeq 6000 platform. Sequencing reads were demultiplexed by UWBC and analyzed using custom-written Python scripts (**Supplementary Note 1**).

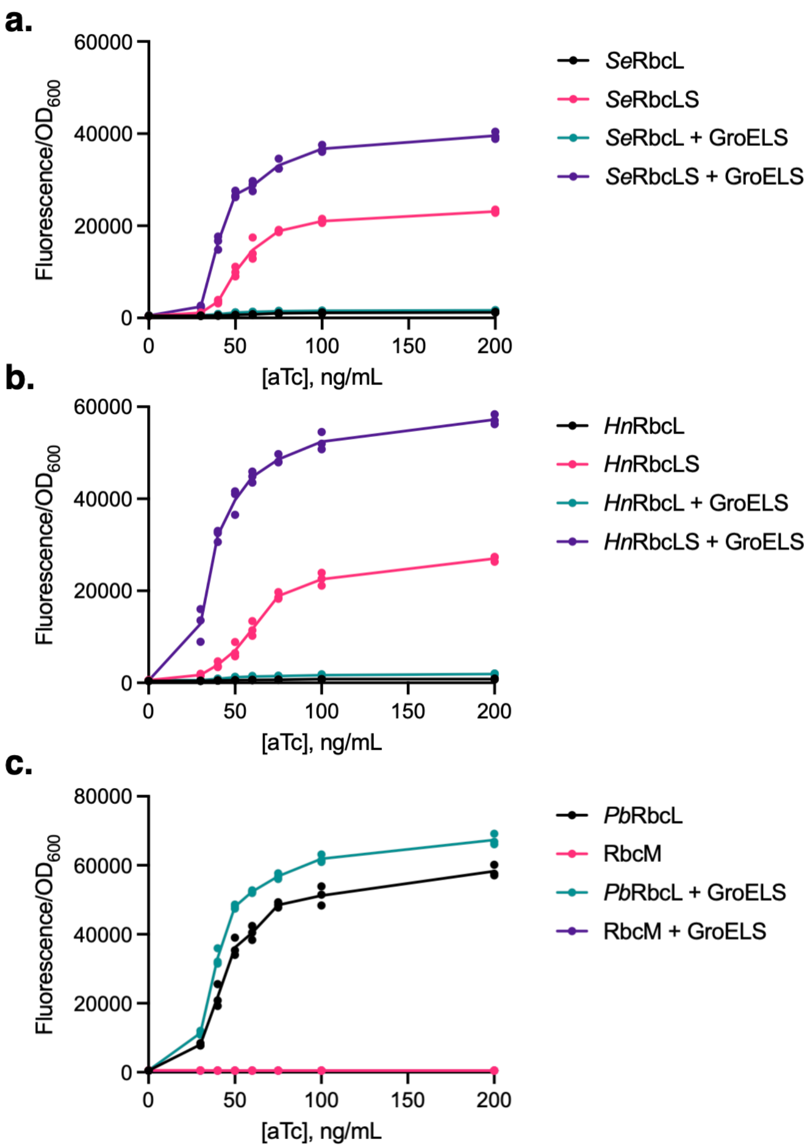

Supplementary Figure 1. (**a-c**) Fluorescence signal generated by GFP expressed by P_{cadBA} upon the induction by aTc of N-terminal fusions of cCadC to RbcL from **(a)** *S. elongatus*, **(b)** *H. neapolitanus*, or **(c)** *P. breve* and *R. rubrum*, normalized by optical density (OD_{600}). GroELS was induced with 0.1 mM IPTG. Where applicable, RbcS was co-expressed with cCadC-RbcL fusions under P_{tet} with its own ribosome binding site.

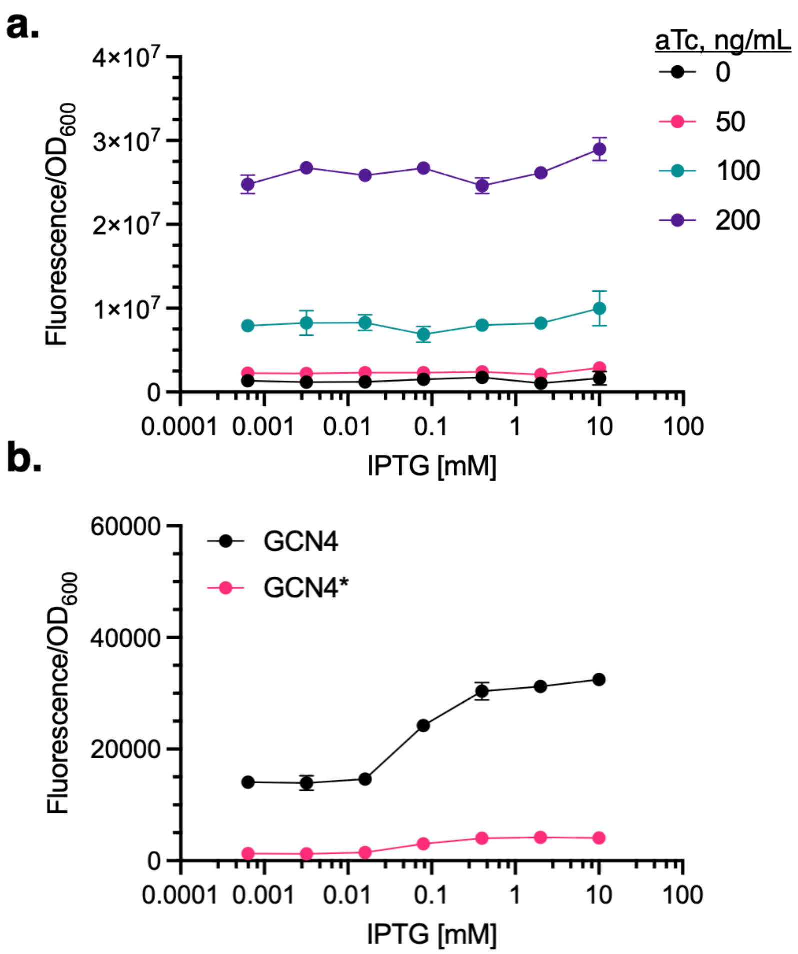

Supplementary Figure 2. (**a**) Effects of increasing IPTG-inducible GroELS expression levels on mGreenLantern (*gfp*) fluorescence. mGreenLantern was induced at varying levels from P_{tet} using aTc. Data was collected using a PerkinElmer EnVision 2105 plate reader. (**b**) Effects of increasing IPTG-inducible GroELS expression levels on the activity of cCadC-GCN4 and cCadC-GCN4* fusions (expressed under P_{tet} and induced with 50 ng/mL aTc). GCN4 forms dimers and GCN4* is a monomeric mutant. The data shown in this figure were generated using strain S2060.

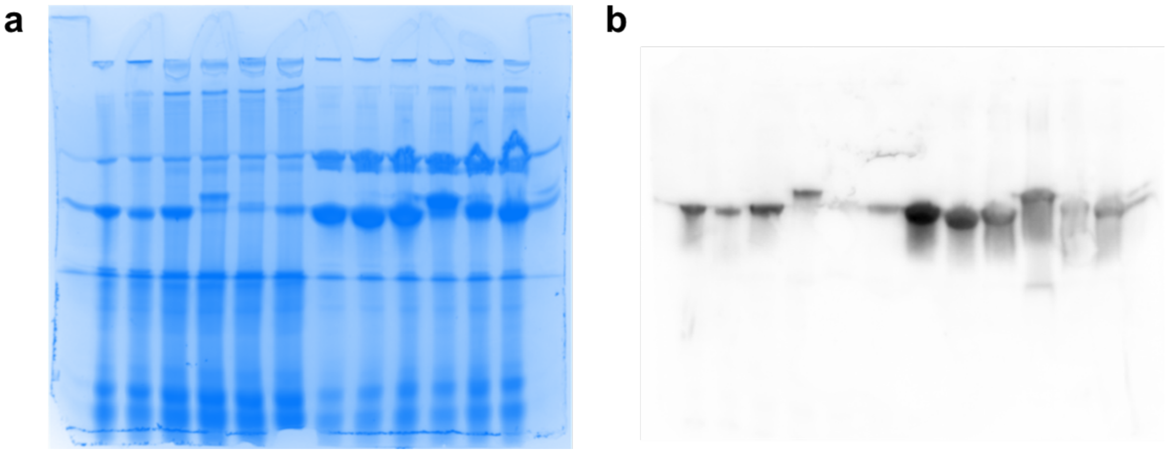

Supplementary Figure 3. Uncropped **(a)** native PAGE gel and **(b)** α-RbcL western blot from **Figure 4**.

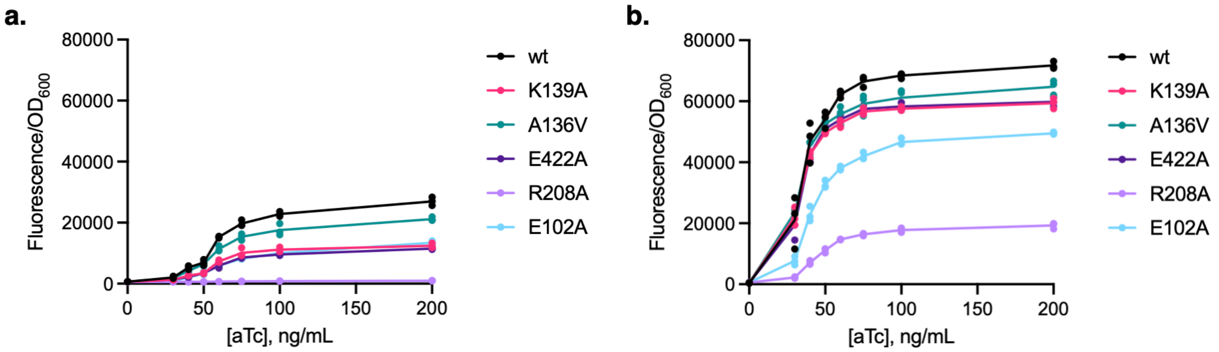

Supplementary Figure 4. Fluorescence signal generated by GFP expressed by P_{cadBA} upon the induction by aTc of N-terminal fusions of cCadC to *Hn*RbcL fusions in the absence **(a)** or presence **(b)** of GroELS overexpression (induced with 0.1 mM IPTG). *Hn*RbcS was co-expressed with cCadC-*Hn*RbcL fusions under P_{tet} with its own ribosome binding site.

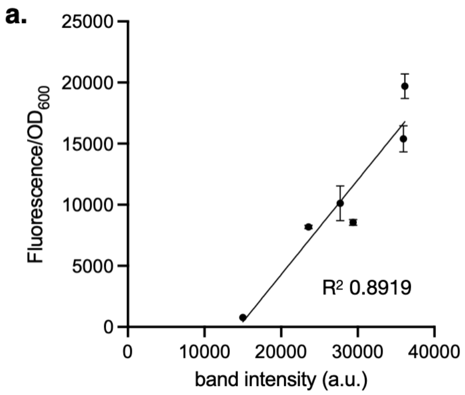

Supplementary Figure 5. Relationship between GFP signal generated by cCadC-*Hn*RbcL mutants (**Figure 3c**) and band intensity of unfused *Hn*Rubisco mutants detected by native PAGE (**Figure 3b**). Panel **(a)** corresponds to samples measured in the absence of GroELS overexpression and panel **(b)** shows samples measures in the presence of GroELS overexpression.

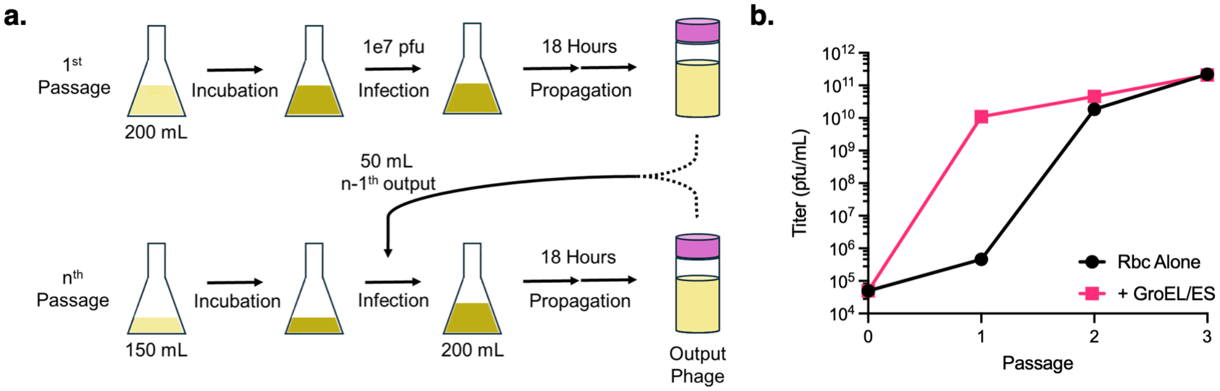

Supplementary Figure 6. (a) *Hn*RbcL library selection scheme. Host cells (S2060 *ΔcadABC*) containing pJH048 (P_{cadBA} *gIII*) and pJW046 (P_{BAD} GroELS) grown to mid-log are infected with 10^7 pfu (plaque-forming units) of phage encoding the *Hn*RbcL library. After 18 hours of propagation, phage are harvested and used to infect host cells in a subsequent passage. **(b)** Phage titers for each passage of selection of the *Hn*RbcL library, with and without GroELS overexpression.

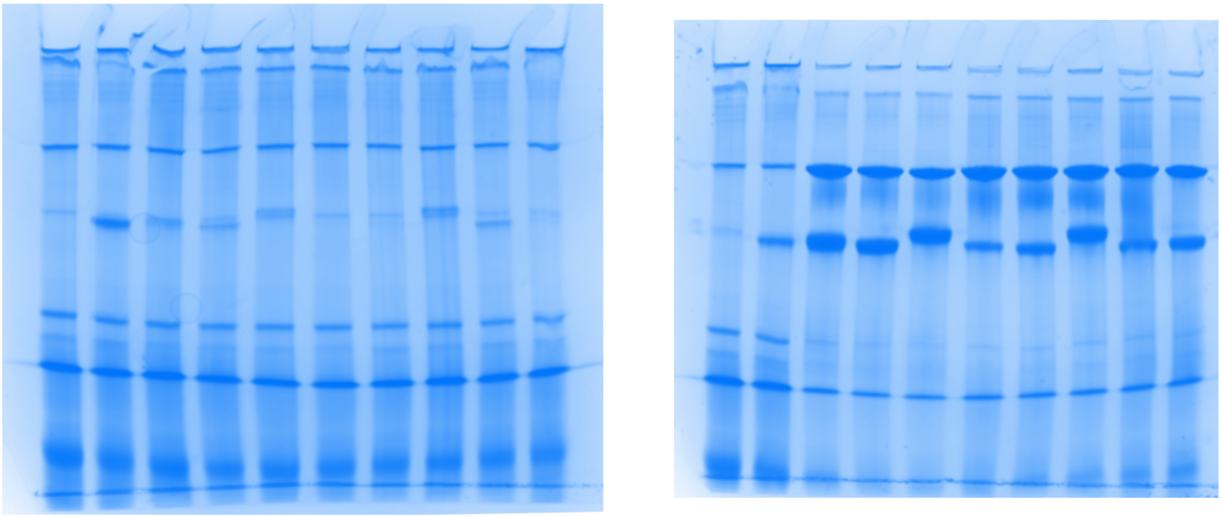

Supplementary Figure 7. Uncroppped native PAGE gels from **Figure 6**.

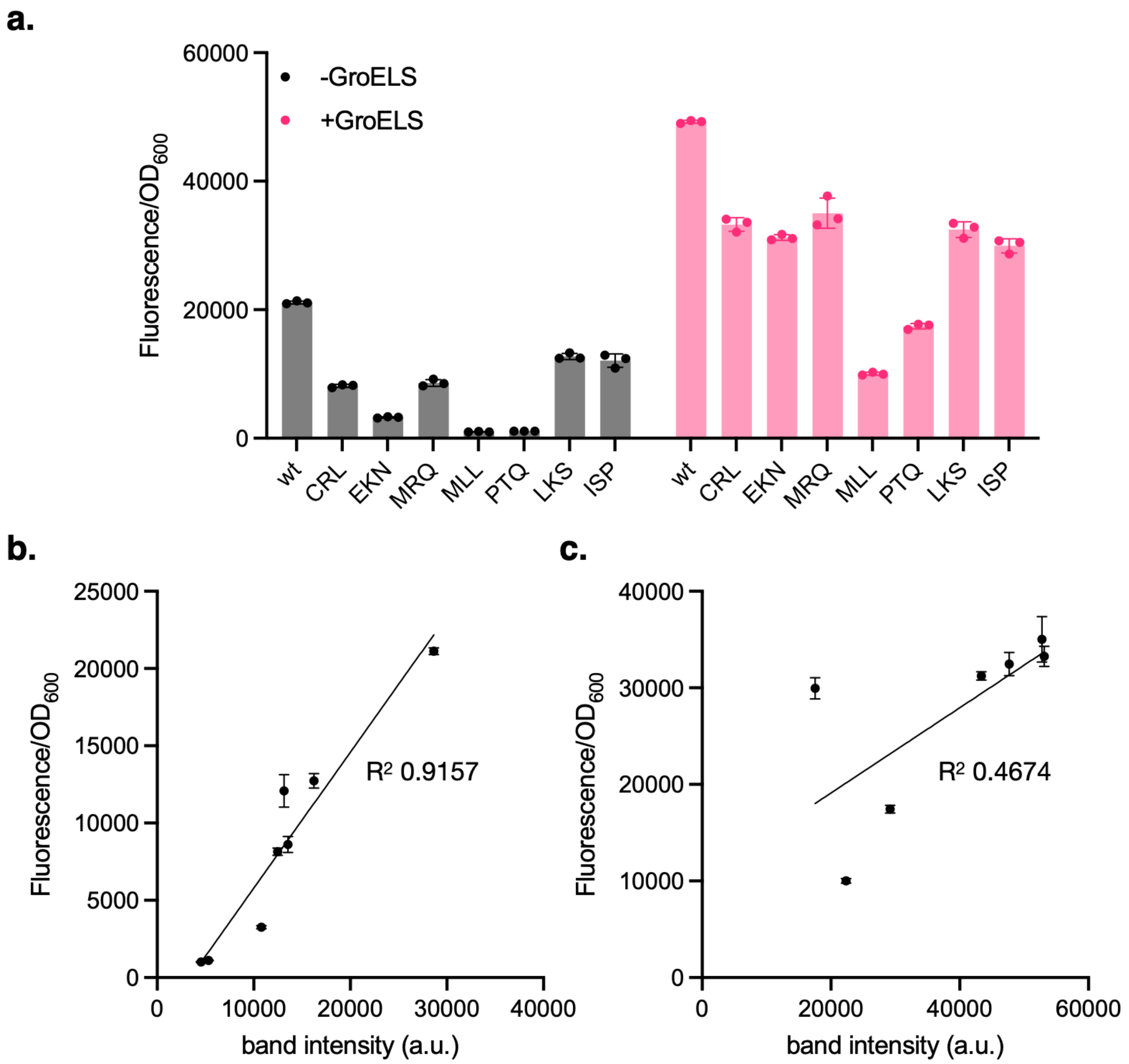

Supplementary Figure 8. (a) Ability of cCadC-*Hn*RbcL variants from PANCS to activate *gfp* transcription from P_{cadBA} in the absence or presence of GroELS overexpression. *Hn*RbcS was co-expressed in this experiment. cCadC fusions were induced at 75 ng/mL aTc and GroELS expression induced at 0.1 mM IPTG. **(b-c)** Relationship between GFP signal generated by cCadC-*Hn*RbcL mutants shown in panel **(a)** and band intensity of unfused *Hn*Rubisco mutants detected by native PAGE (**Figure 6**). Panel **(b)** corresponds to samples measured in the absence of GroELS overexpression and panel **(c)** shows samples measures in the presence of GroELS overexpression.

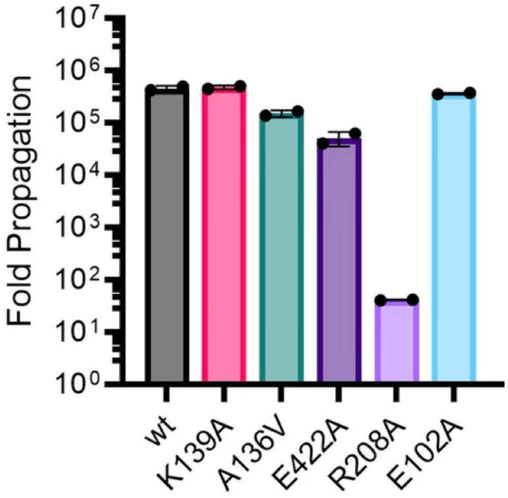

Supplementary Figure 9. Propagation activity of phage encoding *Hn*RbcS and cCadC fusions to wild-type *Hn*RbcL or mutants with varying degrees of assembly on host cells carrying a plasmid supplying *gIII* from P_{cadBA}. Fold propagation is calculated as the number of phage generated from an infected culture divided by the number of phage (10^5) used to infect the culture. This experiment was performed without GroELS overexpression.

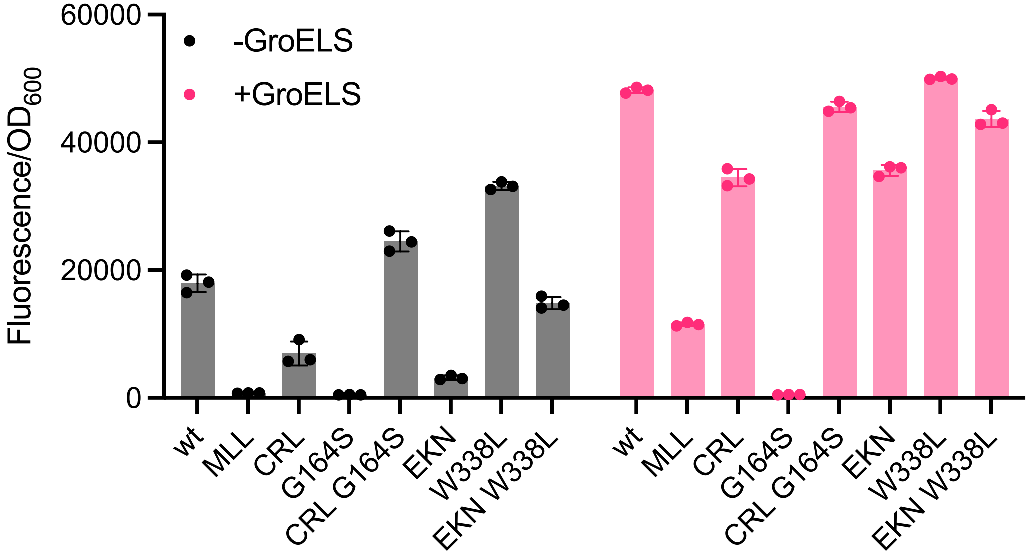

Supplementary Figure 10. Ability of cCadC-*Hn*RbcL variants to activate *gfp* transcription from P_{cadBA}. The MLL variant is an assembly-deficient negative control. *Hn*RbcS was co-expressed in this experiment. cCadC fusions were induced at 75 ng/mL aTc and GroELS expression induced at 0.1 mM IPTG.

Supplementary Table 1. Results from *Hn*RbcL library selection (stop codons excluded). Found in associated Excel file.
Supplementary Table 2. Results from *Hn*RbcL library selection (includes stop codons). Found in associated Excel file.

Supplementary Table 3. Plasmids and phage used in this work.

**Plasmid name**	**Resistance**	**Origin**	**ORF1**		**ORF2**	
			Promoter	Gene(s)	Promoter	Gene(s)
pJC175e[3]	amp	SC101	P_{psp}	*gIII*, x*luxAB*		
pBL155B	chlor	ColE1	P_{tet}	*cCadC-RbcM*		
pJH048	kan	p15a	P_{cadBA}	*gIII*		
pJW004	chlor	ColE1	P_{tet}	*cCadC-SeRbcL*		
pJW005b	chlor	ColE1	P_{tet}	*cCadC-SeRbcLS*		
pJW010b	spec	cloDF13	P_{tac}	*GroES / GroEL*		
pJW012a	chlor	ColE1	P_{tet}	*cCadC-PbRbcL*		
pJW018	spec	cloDF13	P_{tac}	*groES, groEL*	P_{BAD}	*SeRaf1*
pJW019	spec	cloDF13	P_{tac}	*groES, groEL*	P_{BAD}	*SeRbcX*
pJW022	chlor	ColE1	P_{tet}	*cCadC-HnRbcL*		
pJW023a	chlor	ColE1	P_{tet}	*cCadC-HnRbcL, HnRbcS*		
pJW023b	chlor	ColE1	P_{tet}	*cCadC-HnRbcL K139A, HnRbcS*		
pJW023c	chlor	ColE1	P_{tet}	*cCadC-HnRbcL A136V, HnRbcS*		
pJW023d	chlor	ColE1	P_{tet}	*cCadC-HnRbcL E422A, HnRbcS*		
pJW023e	chlor	ColE1	P_{tet}	*cCadC-HnRbcL R208A, HnRbcS*		
pJW023f	chlor	ColE1	P_{tet}	*cCadC-HnRbcL E102A, HnRbcS*		
pJW031	spec	cloDF13	P_{tac}	*groES, groEL*	P_{BAD}	*HnRaf2*
pJW033BA	amp	ColE1	P_{tac}	*HnRbcL, HnRbcS*		
pJW033BB	amp	ColE1	P_{tac}	*HnRbcL K139A, HnRbcS*		
pJW033BC	amp	ColE1	P_{tac}	*HnRbcL A136V, HnRbcS*		
pJW033BD	amp	ColE1	P_{tac}	*HnRbcL E422A, HnRbcS*		
pJW033BE	amp	ColE1	P_{tac}	*HnRbcL R208A, HnRbcS*		
pJW033BF	amp	ColE1	P_{tac}	*HnRbcL E102A, HnRbcS*		
pJW033LA	amp	ColE1	P_{tac}	*HnRbcL D331C R332R A333L, HnRbcS*		
pJW033LB	amp	ColE1	P_{tac}	*HnRbcL D331E R332K A333N, HnRbcS*		
pJW033LC	amp	ColE1	P_{tac}	*HnRbcL D331M R332R A333Q, HnRbcS*		
pJW033LE	amp	ColE1	P_{tac}	*HnRbcL D331M R332L A333L, HnRbcS*		

pJW033LF	amp	ColE1	P_{tac}	*HnRbcL D331P R332T A333Q, HnRbcS*		
pJW033LG	amp	ColE1	P_{tac}	*HnRbcL D331L R332K A333S, HnRbcS*		
pJW033LH	amp	ColE1	P_{tac}	*HnRbcL D331I R332S A333P, HnRbcS*		
pJW039	spec	cloDF13	P_{tac}	*groES, groEL*	P_{BAD}	*AnaRbcX*
pJW040	chlor	ColE1	P_{tet}	*cCadC-SeRbcL ΔC*		
pJW041	chlor	ColE1	P_{tet}	*cCadC-SeRbcL ΔC, SeRbcS*		
pJW046	spec	cloDF13	P_{BAD}	*groES, groEL*		
pSL012	kan	p15a	P_{cadBA}	*mGreenLantern*		
spJW001a	N/A	M13 ΔgIII	P_{gIII}	*cCadC-HnRbcL, HnRbcS*		
spJW001e	N/A	M13 ΔgIII	P_{gIII}	*cCadC-HnRbcL R208A, HnRbcS*		

Supplementary Table 4. Primers used in this work.

Primer Name	5' -> 3' Sequence	Purpose
**JW0112**	actctgggc**U**ggattgatttgctccgcgaatcg	*Hn*RbcL library generation
**JW0113**	agcccagag**U**aga**mnnmnnmnn**gccttccagtttgccgacaa	*Hn*RbcL library generation
**AB019**	agcgctaaa**U**cgggggcc	*Hn*RbcL library generation
**AB020**	atttagcgc**U**tgacggggaa	*Hn*RbcL library generation
**JW0117**	ACACTCTTTCCCTACACGACGCTCTTCCGATCTTTTGTCGGG TGGCGATCAC	*Hn*RbcL primer for Illumina sequencing
**JW0118**	GTGACTGGAGTTCAGACGTGTGCTCTTCCGATCTCGCGAGCG ATCTTCAGGGATAAAC	*Hn*RbcL primer for Illumina sequencing

Supplementary Table 5. General reagents and equipment.

Name	Source	Catalog #	Notes
ampicillin	Gold Biotechnology		
spectinomycin	Gold Biotechnology		
chloramphenicol	Gold Biotechnology		
kanamycin	Gold Biotechnology		
L-arabinose	Gold Biotechnology		
2xYT media	US Biologicals		
LB media	US Biologicals		
agar	US Biologicals		
Davis Rich Media (DRM)	US Biologicals		
Nuclease free water	Omega Bio-Tek		
DTT	DOT scientific		

Isopropyl β-D-1-thiogalactopyranoside	Gold Biotechnology		
X-gal	Gold Biotechnology		
Q5 DNA Polymerase	New England Biolabs	M091S	
Q5U DNA Polymerase	New England Biolabs	M0515S	
USER Enzyme	New England Biolabs	M5505S	
DpnI	New England Biolabs	R0176S	
T4 DNA Ligase	New England Biolabs	M0202S	
T4 PNK	New England Biolabs	M0201S	
T7 DNA Ligase	New England Biolabs	M0318S	
Black clear-bottom 96 well plates	Corning	3881	
96 deep-well plates	VWR	75870-796	
ReadyBlue Protein Gel Stain	Sigma-Aldrich	41105322	
PVDF Membranes	BioRad	1620177	
7.5% TGX protein gel	BioRad	4561025	
40% acrylamide	BioRad	1610140	
B-PER Lysis buffer	ThermoFisher	78243	
Radiance Q chemiluminescence substrate	Azure	AC2101	
rabbit α-rbcL polyclonal antibody	Agrisera	AS03 037	
HRP-conjugated goat α-rabbit secondary antibody	Azure	AC2114	
Tecan Infinite M Plex Plate reader	Tecan		
BioRad Gel-Doc EZ Imager	BioRad		
Azure c400 Gel Imager	Azure		

Supplementary Note 1. Python scripts for analyzing HTS data

```
Script 1:

# takes a fastq file of HTS sequencing, extracts the DNA sequences, trims
them to remove
# sequences outside of the NNK region, converts the list of NNK sequences to
a list of
# amino acid sequences, calculates the number of reads for each sequence, and
finally
# writes it all out into an excel file

# a subset of these scripts originally written by ChatGPT and subsequently
edited by
# the authors

# required packages: matplotlib, pandas, openpyxl

import os
import matplotlib.pyplot as plt
import pandas as pd
from collections import Counter
import csv

# writes a list of sequences to a text file
def write_list_to_file(lst, filename):
    with open(filename, 'w') as file:
        for item in lst:
            file.write("%s\n" % item)

# writes a list of sequences to fasta format
def write_list_to_fasta(lst, filename):
    with open(filename, 'w') as file:
        for i, item in enumerate(lst):
            file.write(">seq%d\n" % i)
            file.write("%s\n" % item)

# takes in a dictionary and a user-defined filename and writes out a text
file
# that contains the dictionary
def write_dict_to_file(dictionary, filename):
    with open(filename, 'w') as f:
        for key, value in dictionary.items():
            f.write(str(key) + ':' + str(value) + '\n')

def dict_to_csv(input_dict, output_filename):
    with open(output_filename, 'w', newline='') as csvfile:
        writer = csv.writer(csvfile)
        # Write the header row
        writer.writerow(['Key', 'Value'])
        # Write the key-value pairs
        for key, value in input_dict.items():
            writer.writerow([key, value])

def reverse_complement(seq):
    complement = {'A': 'T', 'C': 'G', 'G': 'C', 'T': 'A'}
```

```
    return ''.join(complement.get(base, base) for base in reversed(seq))

def reverse_complement_sequences(sequences):
      rc_seq = []
      for i in range(0, len(sequences)):
            rc_seq.append(reverse_complement(sequences[i]))
      return rc_seq

def get_phred_score(c):
    return ord(c) - 33

def is_high_quality(seq, threshold=20):
    high_quality_count = 0
    for c in seq:
        if get_phred_score(c) >= threshold:
            high_quality_count += 1
    return high_quality_count / len(seq) >= 0.95

def filter_sequences_by_quality(fastq_file):
    sequences = []
    with open(fastq_file) as f:
        while True:
            header = f.readline().rstrip()
            if not header:
                break
            seq = f.readline().rstrip()
            f.readline()
            qual = f.readline().rstrip()
            if is_high_quality(qual):
                sequences.append(seq)
    return sequences

# returns list of the DNA sequences from fastq file
def get_seq_list(filename):
      sequences = []
      with open(filename, 'r') as f:
            lines = f.readlines()
            for i in range(0, len(lines), 4):
                  sequences.append(lines[i+1].strip())
      return sequences

# converts fastq file into a dictionary
def read_fastq_file(filename):
    fastq_dict = {}
    with open(filename, 'r') as f:
        lines = f.readlines()
        for i in range(0, len(lines), 4):
            seq_id = lines[i].strip()
            seq = lines[i+1].strip()
            qual_id = lines[i+2].strip()
            qual = lines[i+3].strip()
            fastq_dict[seq_id] = {'seq': seq, 'qual': qual}
    return fastq_dict

#trims sequence starting at a motif and going in the 3' direction so 3' end
is cut off
def trim_sequence_3prime(sequence, motif):
```

```
    pos = sequence.find(motif)
    if pos != -1:
      return sequence[:pos]
    else:
        return sequence

#removes motif starting at a motif and going in the 5' direction so 5' end is
cut off
def trim_sequence_5prime(sequence, motif):
    pos = sequence.find(motif)
    if pos != -1:
        return sequence[pos+len(motif):]
    else:
        return sequence

# takes in a DNA sequence as a string, converts it to a protein sequence, and
returns
# the protein sequence as a string
def translate(sequence):
    genetic_code = {
        'ATA':'I', 'ATC':'I', 'ATT':'I', 'ATG':'M',
        'ACA':'T', 'ACC':'T', 'ACG':'T', 'ACT':'T',
        'AAC':'N', 'AAT':'N', 'AAA':'K', 'AAG':'K',
        'AGC':'S', 'AGT':'S', 'AGA':'R', 'AGG':'R',
        'CTA':'L', 'CTC':'L', 'CTG':'L', 'CTT':'L',
        'CCA':'P', 'CCC':'P', 'CCG':'P', 'CCT':'P',
        'CAC':'H', 'CAT':'H', 'CAA':'Q', 'CAG':'Q',
        'CGA':'R', 'CGC':'R', 'CGG':'R', 'CGT':'R',
        'GTA':'V', 'GTC':'V', 'GTG':'V', 'GTT':'V',
        'GCA':'A', 'GCC':'A', 'GCG':'A', 'GCT':'A',
        'GAC':'D', 'GAT':'D', 'GAA':'E', 'GAG':'E',
        'GGA':'G', 'GGC':'G', 'GGG':'G', 'GGT':'G',
        'TCA':'S', 'TCC':'S', 'TCG':'S', 'TCT':'S',
        'TTC':'F', 'TTT':'F', 'TTA':'L', 'TTG':'L',
        'TAC':'Y', 'TAT':'Y', 'TAA':'*', 'TAG':'*',
        'TGC':'C', 'TGT':'C', 'TGA':'*', 'TGG':'W',
    }
    protein_seq = ''
    if len(sequence) % 3 != 0:
        print('Warning: The sequence length is not a multiple of 3. The last
few nucleotides will be ignored.')
    for i in range(0, len(sequence)-2, 3):
        codon = sequence[i:i+3]
        amino_acid = genetic_code.get(codon, 'X')
        protein_seq += amino_acid
    return protein_seq

# trims the flanking DNA sequences between the two motifs and returns them as
a list
def trim_all(sequences, motif1, motif2):
      trimmed = []
      corrected = []
      for i in range(0,len(sequences)):
            seq = sequences[i]
            trimmed_seq = trim_sequence_3prime(seq,motif1)
            trimmed_seq2 = trim_sequence_5prime(trimmed_seq,motif2)
            trimmed.append(trimmed_seq2)
```

```
	for i in range(0,len(trimmed)):
		if len(trimmed[i]) == 9:
			corrected.append(trimmed[i])
	return corrected

# converts the list of DNA sequences to a list of AA sequences
def get_AA_seq(seq):
	AA_seq = []
	for sequence in seq:
		AA_seq.append(translate(sequence))
	return AA_seq

# takes in a list of AA sequences and returns a list of the unique sequences
def get_unique_seq(plist):
	p = list(plist)
	uplist = []
	for i in range(0, len(p)):
		if p[i] not in uplist:
			uplist.append(p[i])
	return uplist

# takes in a list of AA sequences and counts how many of each sequence there
is
# returns a dictionary where the key is the AA sequence and the value is the
# number of times that sequence occurs
def count_seq_freq(plist):
	p = list(plist)
	pfreq = {}
	for i in range(0, len(p)):
		if p[i] not in pfreq.keys():
			pfreq[p[i]] = 1
		else:
			pfreq[p[i]] += 1
	return pfreq


# --- change these variables to suit your needs -----

# name of fastq file you want to analyze. Do not include the ".fastq"
extension.
fastq_R1_filename = 'F04_S55_L004_R1_001'
fastq_R2_filename = 'F04_S55_L004_R2_001'

# next two variables define the flanking DNA sequences that need to be
trimmed off
# must be in upper case!!
motif_3prime = 'TCTACTCTGGGC' #downstream of target sequence
motif_5prime = 'AAACTGGAAGGC' #upstream of target sequence

# ----------------------------------------------------


filename_R1 = str(fastq_R1_filename + ".fastq")

filename_R2 = str(fastq_R2_filename + ".fastq")

filtered_seq_list_R1 = filter_sequences_by_quality(filename_R1)
```

```
filtered_seq_list_R2 = filter_sequences_by_quality(filename_R2)

rc_seq_list = reverse_complement_sequences(filtered_seq_list_R2)

final_seq = trim_all(filtered_seq_list_R1,motif_3prime,motif_5prime)

final_seq_rc = trim_all(rc_seq_list,motif_3prime,motif_5prime)

joined_final_seq = final_seq + final_seq_rc

residues = get_AA_seq(joined_final_seq)

write_list_to_file(residues, fastq_R1_filename + "_seq.txt")

freq_dict = count_seq_freq(residues)

write_dict_to_file(freq_dict, fastq_R1_filename + "_freq_dict.txt")

dict_to_csv(freq_dict, fastq_R1_filename + "_seq_frequencies.csv")
```

Script 2:

```
# a subset of these scripts originally written by ChatGPT and subsequently
edited by
# the authors

# required packages: matplotlib, pandas, openpyxl

import os
import matplotlib.pyplot as plt
import pandas as pd
import numpy as np
from collections import Counter

# imports the count dictionary text file into a dict
def read_dict_from_file(filename):
    dictionary = {}
    with open(filename, 'r') as f:
        for line in f:
            key, value = line.strip().split(':')
            dictionary[key] = int(value)
    return dictionary

# takes in dictionary and writes it out to an excel file -- generic version
def dict_to_excel(data_dict, filename):
    # Convert the dictionary into a DataFrame where keys are rows and values
are expanded into separate columns
    df = pd.DataFrame.from_dict(data_dict, orient='index')

    # Reset the index to make the dictionary keys a column
    df.reset_index(inplace=True)

    # Rename columns: first column for keys and the rest for values
    df.columns = ['Key'] + [f'Value {i+1}' for i in range(df.shape[1] - 1)]
```

```
    # Write the DataFrame to an Excel file
    df.to_excel(filename, index=False)

# calculates frequency of total reads for each sequence
def convert_to_frequency(count_dictionary):
      dictionary = {}
      total = 0
      for key in count_dictionary:
            total += count_dictionary[key]
      for key in count_dictionary:
            #set detection threshold to 10 reads
            if count_dictionary[key] >= 10:
                  dictionary[key] = count_dictionary[key]/total
      return dictionary

# combine the frequencies for all four passages into one dict
def merge_frequencies(P0, P1, P2, P3):
      dictionary = {}
      for key in P0:
            dictionary[key] = list()
            dictionary[key].append(P0[key])
            if key in P1.keys():
                  dictionary[key].append(P1[key])
            else:
                  dictionary[key].append(0)
            if key in P2.keys():
                  dictionary[key].append(P2[key])
            else:
                  dictionary[key].append(0)
            if key in P3.keys():
                  dictionary[key].append(P3[key])
            else:
                  dictionary[key].append(0)
      return dictionary

# calculates the slope of the frequencies for each sequence across the 4
passages
def calculate_slope(y_values):
    # Define x values as [0, 1, 2, 3]
    x_values = np.array([0, 1, 2, 3])

    # Convert the y_values list to a numpy array
    y_values = np.array(y_values)

    # Calculate the slope using the formula for linear regression (least
squares fit)
    slope = np.polyfit(x_values, y_values, 1)[0]

    return slope

def slope_of_freqs(merge_dict):
      dictionary = {}
      for key in merge_dict:
            slope = calculate_slope(merge_dict[key])
            dictionary[key] = slope
      return dictionary
```

```
def add_slopes(merge_dict):
      for key in merge_dict:
            slope = calculate_slope(merge_dict[key])
            merge_dict[key].append(slope)
      return merge_dict

filename_P0 = "F00_S51_L004_R1_001_freq_dict"
filename_P1 = "F02_S53_L004_R1_001_freq_dict"
filename_P2 = "F04_S55_L004_R1_001_freq_dict"
filename_P3 = "F06_S57_L004_R1_001_freq_dict"

sequence_counts_P0 = read_dict_from_file(filename_P0 + ".txt")
sequence_frequencies_P0 = convert_to_frequency(sequence_counts_P0)

sequence_counts_P1 = read_dict_from_file(filename_P1 + ".txt")
sequence_frequencies_P1 = convert_to_frequency(sequence_counts_P1)

sequence_counts_P2 = read_dict_from_file(filename_P2 + ".txt")
sequence_frequencies_P2 = convert_to_frequency(sequence_counts_P2)

sequence_counts_P3 = read_dict_from_file(filename_P3 + ".txt")
sequence_frequencies_P3 = convert_to_frequency(sequence_counts_P3)

merged_dict = merge_frequencies(sequence_frequencies_P0,
sequence_frequencies_P1, sequence_frequencies_P2, sequence_frequencies_P3)

#slope_dictionary = slope_of_freqs(merged_dict)

add_slopes(merged_dict)

dict_to_excel(merged_dict, "test_slopes.xlsx")
```

Supplementary References

(1) Hubbard, B. P.; Badran, A. H.; Zuris, J. A.; Guilinger, J. P.; Davis, K. M.; Chen, L.; Tsai, S. Q.; Sander, J. D.; Joung, J. K.; Liu, D. R. Continuous Directed Evolution of DNA-Binding Proteins to Improve TALEN Specificity. *Nat Methods* 2015, *12* (10), 939–942.

(2) Miller, S. M.; Wang, T.; Liu, D. R. Phage-Assisted Continuous and Non-Continuous Evolution. *Nat Protoc* 2020, *15* (12), 4101–4127. https://doi.org/10.1038/s41596-020-00410-3.

(3) Carlson, J. C.; Badran, A. H.; Guggiana-Nilo, D. A.; Liu, D. R. Negative Selection and Stringency Modulation in Phage-Assisted Continuous Evolution. *Nat Chem Biol* 2014, *10* (3), 216–222.
